# A small proportion of Talin molecules transmit forces at developing muscle attachments in vivo

**DOI:** 10.1371/journal.pbio.3000057

**Published:** 2019-03-27

**Authors:** Sandra B. Lemke, Thomas Weidemann, Anna-Lena Cost, Carsten Grashoff, Frank Schnorrer

**Affiliations:** 1 Max Planck Institute of Biochemistry, Martinsried, Germany; 2 University of Münster, Institute for Molecular Cell Biology, Münster, Germany; 3 Aix Marseille University, CNRS, IBDM, Marseille, France; King's College London, UNITED KINGDOM

## Abstract

Cells in developing organisms are subjected to particular mechanical forces that shape tissues and instruct cell fate decisions. How these forces are sensed and transmitted at the molecular level is therefore an important question, one that has mainly been investigated in cultured cells in vitro. Here, we elucidate how mechanical forces are transmitted in an intact organism. We studied *Drosophila* muscle attachment sites, which experience high mechanical forces during development and require integrin-mediated adhesion for stable attachment to tendons. Therefore, we quantified molecular forces across the essential integrin-binding protein Talin, which links integrin to the actin cytoskeleton. Generating flies expressing 3 Förster resonance energy transfer (FRET)-based Talin tension sensors reporting different force levels between 1 and 11 piconewton (pN) enabled us to quantify physiologically relevant molecular forces. By measuring primary *Drosophila* muscle cells, we demonstrate that *Drosophila* Talin experiences mechanical forces in cell culture that are similar to those previously reported for Talin in mammalian cell lines. However, in vivo force measurements at developing flight muscle attachment sites revealed that average forces across Talin are comparatively low and decrease even further while attachments mature and tissue-level tension remains high. Concomitantly, the Talin concentration at attachment sites increases 5-fold as quantified by fluorescence correlation spectroscopy (FCS), suggesting that only a small proportion of Talin molecules are mechanically engaged at any given time. Reducing Talin levels at late stages of muscle development results in muscle–tendon rupture in the adult fly, likely as a result of active muscle contractions. We therefore propose that a large pool of adhesion molecules is required to share high tissue forces. As a result, less than 15% of the molecules experience detectable forces at developing muscle attachment sites at the same time. Our findings define an important new concept of how cells can adapt to changes in tissue mechanics to prevent mechanical failure in vivo.

## Abstract 2

The protein Talin links the transmembrane cell adhesion molecule integrin to the actin cytoskeleton. Quantitative FRET-based force measurements across Talin in vivo reveal that only few Talin molecules are under force during the development of muscle attachment sites.

## Author summary

Cells in our body are constantly exposed to mechanical forces, which they need to sense and react to. In previous studies, fluorescent force sensors were developed to demonstrate that individual proteins in adhesion structures of a cell experience forces in the piconewton (pN) range. However, these cells were analyzed in isolation in an artificial plastic or glass environment. Here, we explored forces on adhesion proteins in their natural environment within a developing animal and used the muscle–tendon tissue in the fruit fly *Drosophila* as a model system. We made genetically modified fly lines with force sensors or controls inserted into the gene that produces the essential adhesion protein Talin. Using these force sensor flies, we found that only a small proportion of all the Talin proteins (<15%) present at developing muscle–tendon attachments experience detectable forces at the same time. Nevertheless, a large amount of Talin is accumulated at these attachments during fly development. We found that this large Talin pool is important to prevent rupture of the muscle–tendon connection in adult flies that produce high muscle forces during flight. In conclusion, we demonstrated that a large pool of Talin proteins is required for stable muscle–tendon attachment, likely with the individual Talin molecules dynamically sharing the mechanical load.

## Introduction

The shape of multicellular organisms critically depends on the presence of mechanical forces during development [1,2]. Forces not only generate form and flows within tissues [3,4] but can also control cell fate decisions [5,6] and trigger mitosis [7]. There are various ways to quantify forces at the cellular or tissue level [8,9]; however, mechanical forces experienced by proteins in cells have only recently become quantifiable with the development of Förster resonance energy transfer (FRET)-based molecular tension sensors [10]. These sensors contain a donor and an acceptor fluorophore connected by a mechanosensitive linker peptide, which reversibly unfolds and extends when experiencing mechanical forces. As a result, such sensors report forces as a decrease in FRET efficiency caused by an increase in distance between the fluorophores. Since previous studies analyzed molecular forces using in vitro cell culture systems [11–17] and insights from in vivo experiments are still limited [18–21], it remains largely open how mechanical loads are processed at the molecular level in tissues of living organisms.

Integrins are a major and highly conserved force-bearing protein family. They connect the actomyosin cytoskeleton to the extracellular matrix and are essential for numerous mechanically regulated processes in vivo or in vitro [22,23]. However, in vivo it is particularly unclear how integrin-based structures are mechanically loaded because forces have so far only been analyzed in focal adhesions, which are typically not found in soft tissues [11–13,17]. Therefore, we chose to investigate *Drosophila* muscle attachment sites in vivo, which experience high mechanical forces during development [24] and depend on integrin-based attachment of muscle fibers to tendon cells [22,25]. For the molecular force measurements, we selected the integrin activator and mechanotransducer Talin, which is essential for all integrin-mediated functions and binds with its globular head domain to the tail of β-integrin and with its rod domain to actin filaments [26,27]. Thus, Talin is in the perfect position to sense mechanical forces across integrin-dependent adhesive structures. In contrast to measurements performed previously in vitro [12], we find that less than 15% of the Talin molecules experience significant forces at developing muscle attachments in vivo, suggesting that high tissue forces are sustained by recruiting a large excess of Talin molecules to muscle attachments. Reducing the Talin levels leads to rupture of muscle attachments in response to high forces during adult muscle contractions. This demonstrates the significance of high Talin levels for the robustness of muscle attachments under peak mechanical load.

## Results

### A *Drosophila* Talin tension sensor

**Fig 1:**
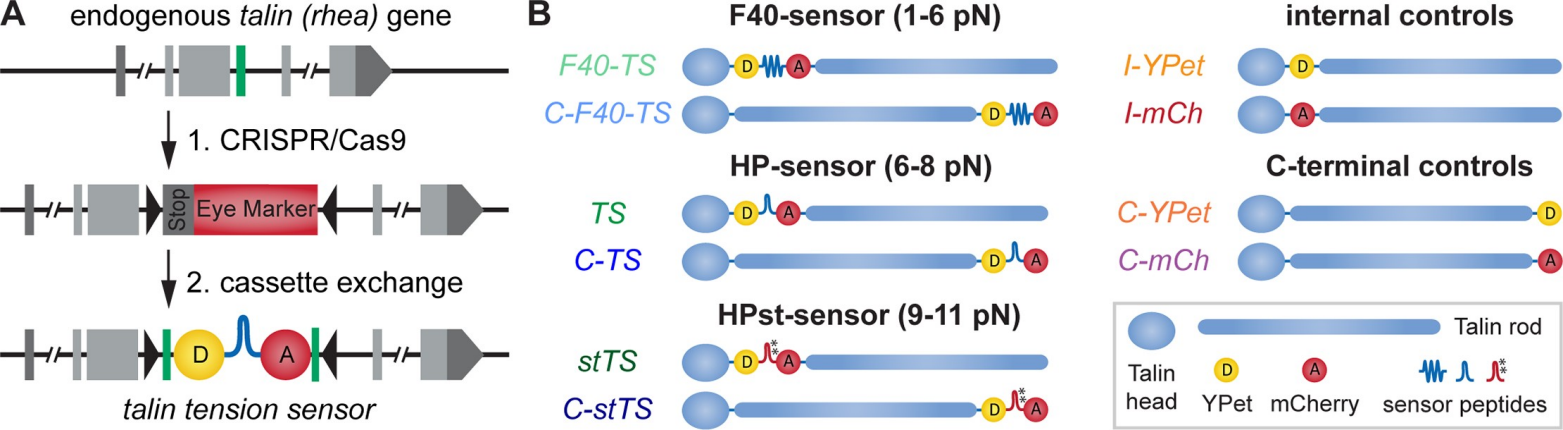
Talin tension sensor generation. (A) Two-step genome engineering strategy of the *talin* (*rhea*) gene. Step 1: Cas9-mediated insertion of an eye marker cassette replacing the target exon (green). Step 2: ϕC31-mediated cassette exchange restoring the original exon and including a tension sensor. See S1 Fig for details. (B) Overview of Talin tension sensor and control flies. Sensors with 3 different mechanosensitive linker peptides, F40, HP, and HPst, were generated. Respective force regimes are indicated. Each sensor was inserted internally (F40-TS, TS, stTS) or at the C-terminus (C-F40-TS, C-TS, C-stTS). Controls with the individual fluorescent proteins were also generated (I-YPet, I-mCh, C-YPet, C-mCh). C-F40-TS, Talin control sensor with F40-sensor module; C-mCh, Talin with C-terminal mCherry; C-stTS, Talin control sensor with HPst-sensor module; C-TS, Talin control sensor with HP-sensor module; C-YPet, Talin with C-terminal YPet; CRISPR/Cas9, clustered regularly interspaced short palindromic repeats/CRISPR-associated protein 9; F40, Flagelliform peptide; F40-TS, Talin tension sensor with F40-sensor module; HP, Villin headpiece; HPst, stable Villin headpiece; I-mCh, Talin with internal mCherry; I-YPet, Talin with internal YPet; pN, piconewton; stTS, Talin tension sensor with HPst-sensor module; TS, Talin tension sensor with HP-sensor module; YPet, yellow fluorescent protein for energy transfer.

To enable quantitative force measurements, we generated various *Drosophila* Talin tension sensor and control flies by modifying the endogenous *talin* (*rhea*) gene using a two-step strategy based on clustered regularly interspaced short palindromic repeats (CRISPR)/CRISPR-associated protein 9 (Cas9) genome engineering and ϕC31-mediated cassette exchange (Fig 1A, S1 Fig) [28]. This strategy enabled us to generate an entire set of Talin tension sensor fly lines with yellow fluorescent protein for energy transfer (YPet) and mCherry FRET pairs and 3 different mechanosensitive linker peptides [11,13], Flagelliform (F40), Villin headpiece peptide (HP), and HP’s stable variant (HPst), reporting forces of 1–6 piconewton (pN), 6–8 pN, and 9–11 pN, respectively (Fig 1B). The sensor modules were inserted both internally between the Talin head and rod domains (F40-TS, TS, stTS) at the analogous position used in mammalian Talin to report forces in vitro [11,17] and C-terminally as a zero-force control (C-F40-TS, C-TS, C-stTS). Furthermore, the individual fluorescent proteins were inserted at both positions as controls (I-YPet, I-mCh, C-YPet, C-mCh). All stocks are homozygous viable and fertile and do not display any overt phenotype indicating that the Talin tension sensor proteins are functional.

**Fig 2:**
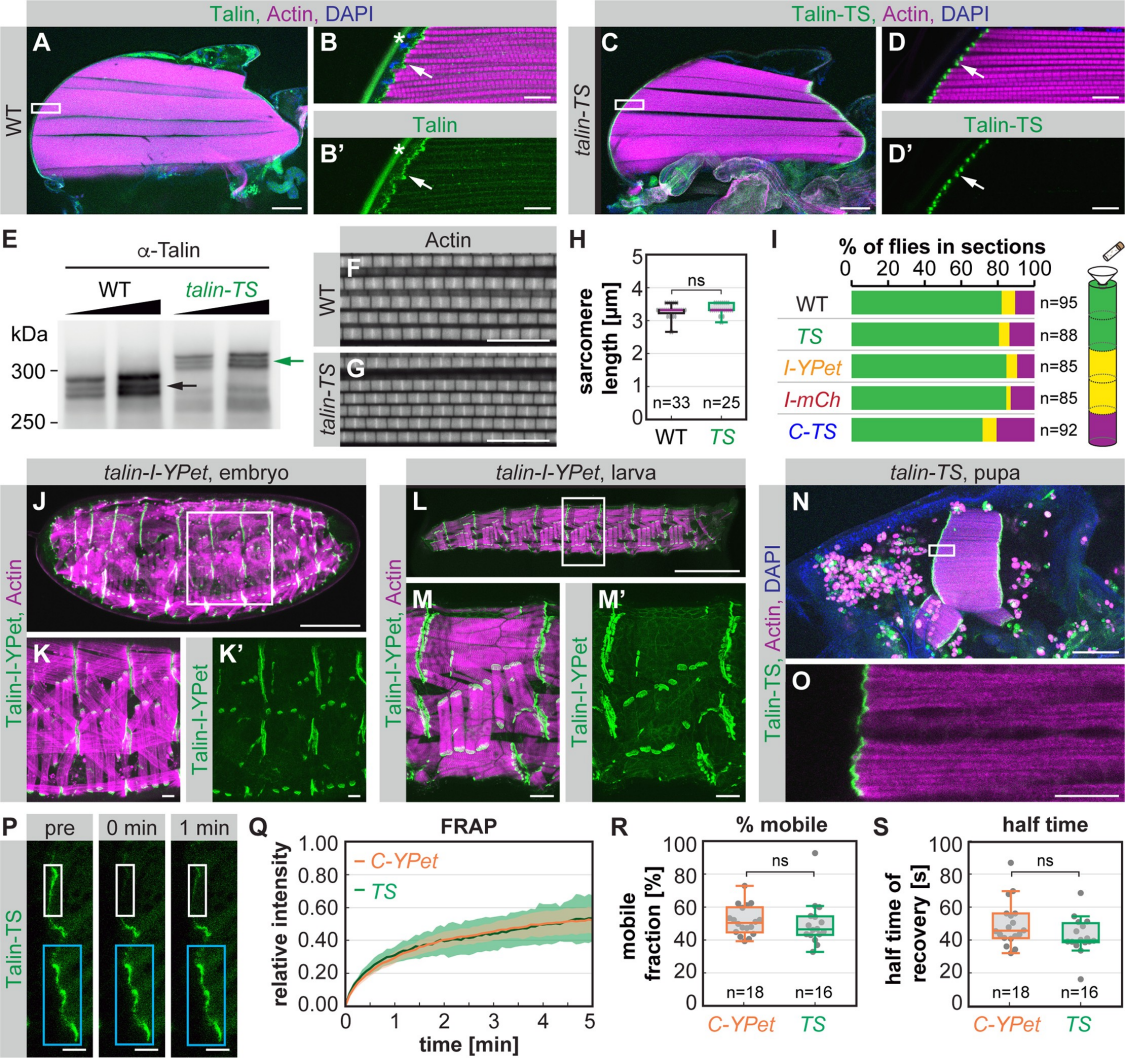
Verification of Talin tension sensor protein functionality. (A–B) WT adult hemithorax stained with Talin antibody, phalloidin (actin), and DAPI. White box in A indicates zoom-in area shown in B and B’. Note the Talin localization at myofibril tips (arrow). The star indicates background fluorescence from the cuticle. (C–D) *talin-TS* adult hemithorax showing Talin-TS localization at myofibril tips (arrow). (E) Western blot of whole fly extract from WT and *talin-TS* flies probed with Talin antibody. Note the up-shift of all Talin-TS bands (green arrow) compared to WT (black arrow). (F–H) Phalloidin stainings of adult hemithoraxes showing normal sarcomere morphology in WT (F) and *talin-TS* (G) flies, and normal sarcomere length (H) (Mann Whitney test, ns: *p* > 0.05). (I) Flight test (two-way ANOVA, no significant differences compared to WT in 6 replicates). (J–O) Talin-I-YPet or Talin-TS expression at different stages of development. Live images of a stage 17 *talin-I-YPet* embryo (J–K) and an L3 larva (L–M) co-expressing *Mef2-*GAL4, *UAS-*mCherry-Gma as a muscle actin marker. (Because the actin marker contains mCherry, we used Talin-I-YPet here to avoid signal overlap in the mCherry channel.) A 32 h APF *talin-TS* pupa (N–O) stained with phalloidin and DAPI. (P–S) Talin dynamics analyzed by FRAP at flight muscle attachment sites in 24 h APF pupae. Fluorescence intensity was followed in a bleached region (white boxes in P) in comparison to a control region (cyan boxes in P). Time point 0 is directly after bleaching. Talin-TS shows the same recovery dynamics as C-YPet (Q, mean and standard deviation). The mobile fraction (R) and the half time of recovery (S) are indistinguishable (Kolmogorov-Smirnov test, ns: *p* > 0.05). Scale bars are 100 μm in A, C, J, M, and N, 10 μm in B, D, F, G, K, O, and P, and 1 mm in L. Underlying data can be found in S1 Data. C-TS, Talin control sensor with HP-sensor module; C-YPet, Talin with C-terminal YPet; FRAP, fluorescence recovery after photobleaching; h APF, hours after puparium formation; HP, Villin headpiece; I-mCh, Talin with internal mCherry; I-YPet, Talin with internal YPet; ns, not significant; TS, Talin tension sensor with HP-sensor module; WT, wild type; YPet, yellow fluorescent protein for energy transfer.

To assess the functionality of Talin-TS more rigorously, we first analyzed Talin-TS localization in adult hemithoraxes and found that Talin-TS localizes to myofibril tips as expected (Fig 2A–2D). Second, we performed western blot analysis and found the expected band shifts for tension sensor module incorporation into Talin protein isoforms (Fig 2E). Third, we quantified sarcomere length in flight muscles and found the expected length of 3.2 μm in wild-type (WT) [29] and *talin-TS* flies (Fig 2F–2H). Fourth, we tested flight ability [30] and found that neither the insertion of the sensor module nor the individual fluorescent proteins into the internal position nor the insertion of the sensor module at the C-terminus causes flight defects (Fig 2I). Fifth, we confirmed that Talin-TS (or Talin-I-YPet) is expressed correctly at all developmental stages (embryo, larva, and pupa) and is detected most prominently at muscle attachment sites as previously reported for endogenous Talin (Fig 2J–2O) [31]. Finally, we assessed the molecular dynamics of Talin-TS at flight muscle attachments using fluorescence recovery after photobleaching (FRAP). We compared the internal tension sensor to Talin-C-YPet, which is tagged at a functionally verified position [32], and found that internal tagging of Talin does not alter its molecular dynamics. Both the mobile fraction as well as the recovery half time are indistinguishable from C-terminally tagged Talin (Fig 2P–2S). Together, these data demonstrate that the tension sensor module is properly incorporated into Talin and the resulting protein is functional. Thus, Talin-TS is suitable for the quantification of mechanical tension across Talin in any tissue and at any developmental stage of *Drosophila* in vivo.

### Forces across *Drosophila* Talin in primary muscle fiber cultures

**Fig 3:**
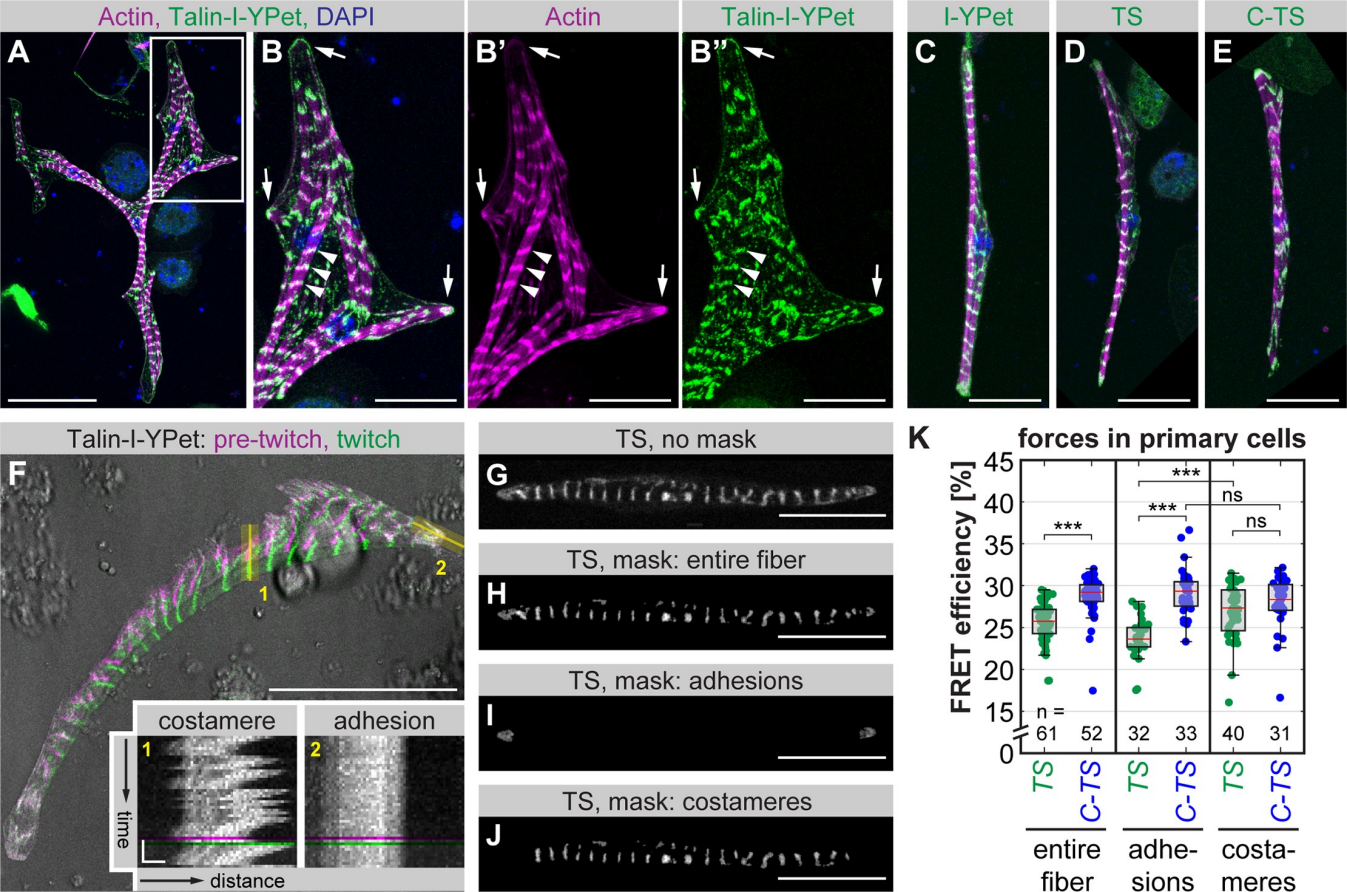
Talin-TS reveals forces in primary muscle fibers. (A–B) Primary myoblasts isolated from *talin-I-YPet* embryos were differentiated and stained with phalloidin and DAPI on day 6. White box in A indicates zoom-in area in B. In differentiated muscle fibers, Talin-I-YPet localizes to adhesions at fiber tips (arrows) and to costameres along myofibrils (arrowheads). (C–E) Primary muscle fibers differentiated from *talin-I-YPet* (C), *talin-TS* (D), or *talin-C-TS* (E) embryos stained with phalloidin (magenta) and DAPI (blue) show similar morphologies and Talin localization (green). (F) Transmission light image (grey) of a twitching primary muscle cell overlaid with Talin-I-YPet signal pre-twitch (magenta) and during the twitch (green), and kymographs of the regions indicated in yellow. Note that costameres move with contractions, while adhesions are fixed to the substrate. See S1 Movie. (G–J) Masking of cells for force analysis. From the original image (G), masks from the entire fiber (H), from adhesions at fiber tips (I), or from costameres (J) were created. (K) Talin forces measured by FLIM-FRET. A decrease in FRET efficiency of Talin-TS (TS) compared to the C-terminal zero-force control (C-TS) indicates force. Note that Talin in adhesions but not in costameres experiences a significant amount of force (Kolmogorov-Smirnov test, ***p < 0.001, ns: *p* > 0.05). Scale bars are 50 μm in A and F and 20 μm in B–E and G–J. Scale bars in kymographs in F are 10 s and 2 μm. Underlying data can be found in S1 Data. C-TS, Talin control sensor with HP-sensor module; FLIM, fluorescence lifetime imaging microscopy; FRET, Förster resonance energy transfer; HP, Villin headpiece; I-YPet, Talin with internal YPet; ns, not significant; TS, Talin tension sensor with HP-sensor module; YPet, yellow fluorescent protein for energy transfer.

To ensure that our approach is comparable to previous Talin force measurements in cultured mammalian cells, we established muscle fiber cultures by incubating primary myoblasts in vitro for 5 to 7 d [33,34]. Isolated myoblasts from *talin-I-YPet* embryos differentiated into striated, often multinucleated muscle fibers and efficiently adhered to the underlying plastic substrate (Fig 3A and 3B). In these cells, Talin-I-YPet localizes to adhesions at the fiber tips and at myofibril ends as well as to costameres, which connect myofibrils at the sarcomeric Z-discs to the cell membrane [35]. Primary muscle fibers generated from *talin-I-YPet*, *talin-TS*, and *talin-C-TS* embryos display similar morphologies (Fig 3C–3E) and contract spontaneously (S1 Movie). Adhesions at the fiber tips do not move during these contractions, whereas costameres are mobile and thus are not fixed to the plastic substrate (Fig 3F).

For establishing force measurements using these primary fiber cultures, we performed fluorescence lifetime imaging microscopy (FLIM) to determine the FRET efficiency of the Talin tension sensor containing the HP-sensor module (TS) compared to the zero-force control (C-TS). We created distinct masks for Talin FRET signals either in the entire fiber or specifically in cell-substrate adhesions at the fiber tips or in costameres along myofibrils (Fig 3G–3J). Consistent with previous Talin force measurements in cultured fibroblasts [11,17], we observed a reduction in FRET efficiency of TS compared to the control C-TS within the entire fiber, indicating that Talin indeed experiences mechanical forces in these adherent, primary muscle fibers (Fig 3K). As expected, we find higher average forces across Talin at muscle-substrate adhesions compared to the rest of the cell. In costameres, which are not fixed to the plastic substrate, the FRET efficiency of TS is indistinguishable from the control, indicating that forces across Talin at costameres are lower and do not exceed 6 to 8 pN (Fig 3K). Together, these data demonstrate that the *Drosophila* Talin-TS reports similar Talin forces at adhesions of cultured muscle fibers as were previously described for Talin in focal adhesions of mammalian fibroblasts [11,12,17].

### Tissue forces during *Drosophila* muscle–tendon development in vivo

**Fig 4:**
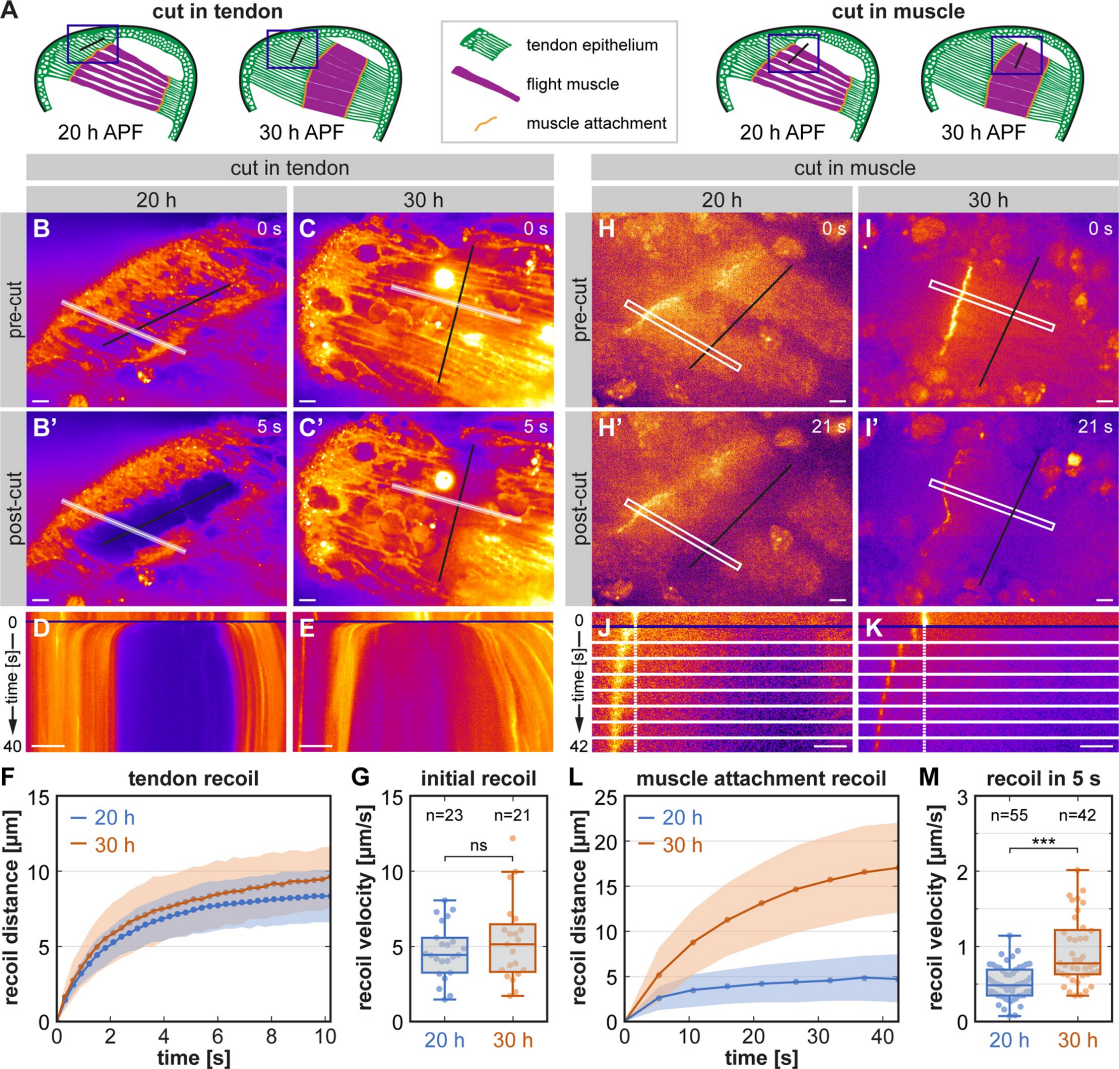
The muscle–tendon system is exposed to high tissue tension during development. (A) Schemes of developing flight muscles in the pupal thorax at 20 h and 30 h APF. Blue boxes indicate the areas imaged during the laser cutting experiments. Black lines indicate the positioning of the laser cuts either in the tendon epithelium or the muscle. (B–E) Stills from movies of *stripe-*GAL4, *Mef2-*GAL4, *UAS-*brainbow pupae expressing palmitoylated mCherry in the tendon and muscle tissue (B–C). B’ and C’ show the tissue recoil after laser cutting the tendon tissue (black line). White lines in B and C indicate areas analyzed in kymographs in D and E highlighting the tissue recoil. Time resolution is 300 ms. See S2 and S3 Movies. (F–G) Quantification of the tendon tissue recoil (F; mean as dots and standard deviation as shaded area) and the initial recoil velocity calculated from the first 2 frames after the cut (G). (Kolmogorov-Smirnov test, ns: *p* > 0.05). (H–K) Stills from movies of pupae expressing Talin-I-YPet as a marker for muscle attachment sites (H–I). H’ and I’ show the recoil of the muscle attachment after laser cutting the muscle in a 10-μm–thick z-stack (black line). White boxes in H and I indicate areas shown in a time course in J and K. Dashed white lines mark the position of the muscle attachments before the cut. Time resolution is 5.3 s. See S6 and S7 Movies. (L–M) Quantification of the muscle attachment recoil (L; mean as dots and standard deviation as shaded area) and the recoil velocity calculated form the pre-cut image and the first frame after the cut (M). (Kolmogorov-Smirnov test, ***p < 0.001). Scale bars are 10 μm. Underlying data can be found in S1 Data. h APF, hours after puparium formation; I-YPet, Talin with internal YPet; ns, not significant; YPet, yellow fluorescent protein for energy transfer.

To quantify forces across Talin in vivo, we chose the developing muscle–tendon attachments of the flight muscles as a model system, which critically depend on integrin and Talin function [24,31]. At 20 hours after puparium formation (h APF), the developing myotubes have initiated contact with the tendon epithelium, and immature muscle attachment sites are formed (Fig 4A). While they mature, the myotubes compact and the tendon epithelium forms long cellular extensions. By 30 h APF, the myotubes have reached their maximally compacted stage (Fig 4A) and have initiated myofibrillogenesis. Thereafter, the muscles elongate and grow to fill the entire thorax by the end of the pupal stage [29]. Previous studies using laser-induced microlesions in developing tendons had shown that increasing mechanical tension is built up in the muscle–tendon tissue from 18 h to 22 h APF and that this tension is required for ordered myofibrillogenesis [24,36]. However, tissue tension at the maximally compacted stage of the muscle fibers at 30 h APF had not been analyzed yet. Therefore, we cut the tendon cells at 20 h and 30 h APF and performed time-lapse imaging to quantify the tendon tissue recoil. As a proxy for tissue tension, we calculated the initial recoil velocity from the first 2 frames after the cut (300 ms) and found that it remains high at 30 h APF (Fig 4B–4G, S2 Movie and S3 Movie).

To ensure that the high tissue tension is also present in the muscle fibers, we cut the muscle at 20 h and 30 h APF (Fig 4A). Cutting the muscle fibers in a single focal plane is not sufficient to cut the entire fiber in two. However, laser lesions in the muscle induce muscle contractions at 30 h APF but not at 20 h APF (S2 Fig, S4 Movie and S5 Movie). This demonstrates that the immature myofibrils present at 30 h APF are contractile and are stably connected to muscle attachments. A similar observation was made before in *Drosophila* abdominal muscles, in which laser-induced lesions cause a Ca^2+^ pulse that triggers contraction of the immature myofibrils [37]. To sever the entire muscle fibers, we cut repeatedly in a 10-μm–thick z-stack and tracked the recoil of the muscle attachments at 20 h and 30 h APF (Fig 4H–4L, S6 Movie and S7 Movie). Due to the z-stack acquisition, our time resolution was limited to 5 s, and therefore we could not determine the initial recoil velocity precisely. Instead, we quantified the average recoil velocity in the first 5 s and found that it increases from 20 h to 30 h APF, suggesting an overall increase in muscle fiber tension between 20 h and 30 h APF (Fig 4M). In conclusion, tissue tension in the muscle–tendon system remains high and possibly increases further from 20 h to 30 h APF, both in the tendon and the muscle tissue.

### Forces across *Drosophila* Talin in vivo

**Fig 5:**
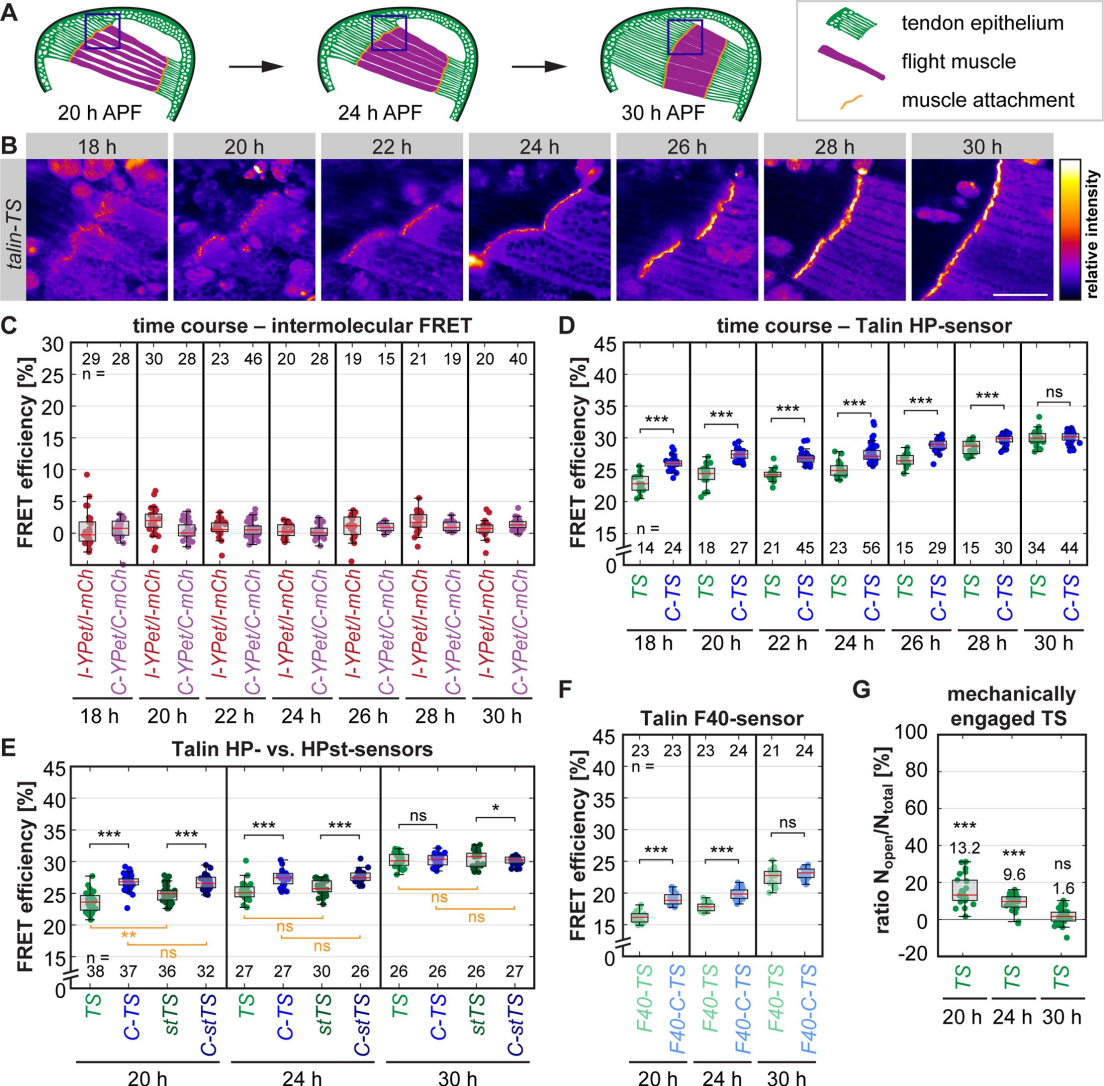
A small proportion of Talin molecules at muscle attachment sites in vivo are mechanically engaged. (A) Schemes of flight muscle development in the pupal thorax at 20, 24, and 30 h APF. Blue boxes indicate areas imaged for force measurements (see B). (B) Images showing Talin-TS localization to maturing muscle attachment sites. Scale bar is 50 μm. (C) Intermolecular FRET control data measured by FLIM-FRET in a time course comparing heterozygous I-YPet/I-mCh or C-YPet/C-mCh pupae to homozygous I-YPet or C-YPet pupae (set to 0), respectively. Intermolecular FRET is negligible at all time points. (D) Talin forces measured by FLIM-FRET using the HP-sensor module (6–8 pN). A decrease in FRET efficiency of Talin-TS compared to the C-terminal zero-force control (C-TS) indicates force. Note that the average force per molecule is highest in the beginning of the time course. (E) Comparisons of TS (6–8 pN) and stTS (9–11 pN) to the C-terminal zero-force controls, C-TS and C-stTS. Note that both sensors indicate forces across Talin at 20 h and 24 h APF (significance indicated in black). Direct comparisons between TS and stTS or the controls are indicated in orange. Note the increase in FRET of stTS compared to TS at 20 h APF. (F) Talin force measurements using the F40-sensor module (1–6 pN). (G) Proportion of mechanically engaged TS determined as the ratio of open (*N*_open_) versus total (*N*_total_) sensor using biexponential fitting. Significance is indicated in comparison to zero-force control level (set to 0). The raw data are the same as in D. (Kolmogorov-Smirnov test, ***p < 0.001, **p < 0.01, *p < 0.05; ns: *p* > 0.05). Underlying data can be found in S1 Data. C-mCh, Talin with C-terminal mCherry; C-stTS, Talin control sensor with HPst-sensor module; C-TS, Talin control sensor with HP-sensor module; C-YPet, Talin with C-terminal YPet; F40, Flagelliform peptide; F40-C-TS, Talin control sensor with F40-sensor module; F40-TS, Talin tension sensor with F40-sensor module; FLIM, fluorescence lifetime imaging microscopy; FRET, Förster resonance energy transfer; F40, Flagelliform peptide; h APF, hours after puparium formation; HP, Villin headpiece; HPst, stable Villin headpiece; I-mCh, Talin with internal mCherry; I-YPet, Talin with internal YPet; ns, nonsignificant; pN, piconewton; stTS, Talin tension sensor with HPst-sensor module; TS, Talin tension sensor with HP-sensor module; YPet, yellow fluorescent protein for energy transfer.

After establishing that tissue forces build up in the muscle–tendon system and remain high until 30 h APF, we measured Talin forces between 18 h and 30 h APF in living pupae at the anterior muscle attachment sites of the dorsal-longitudinal flight muscles using the HP-sensor module (Fig 5A and 5B and workflow in S3 Fig). For calculating the FRET efficiency, we determined the fluorescence lifetime of only the donor in flies expressing YPet at the internal position of Talin (S4A Fig). In addition, we excluded the possibility that FRET between neighbouring molecules (intermolecular FRET) affects our measurements throughout the entire time course (Fig 5C) and confirmed that our lifetime measurements are independent of signal intensity (S4B Fig). We noted that the FRET efficiency of the zero-force control sensor slightly increases over the time course, possibly because the increasing crowding at the attachments restricts the conformational freedom of the sensor and thus may favor FRET (Fig 5D). Therefore, we measured the FRET efficiency of the control sensor in addition to the tension sensor at all developmental time points. In this way, we detected a significant drop in FRET efficiency for Talin-TS compared to the control Talin-C-TS at 18 to 28 h APF (Fig 5D). The FRET efficiency reduction at muscle attachment sites was significantly smaller compared to the in vitro measurements of cultured muscle fibers (Fig 3K) or of cultured mammalian fibroblasts [11]. At 30 h APF, no difference in FRET efficiencies was detected, suggesting that there is little or no tension across Talin at this time point. Together, these data suggest that only a small percentage of Talin molecules at muscle attachments experience forces above 6 pN at 18 to 28 h APF. The remaining molecules could either bear no force or forces below 6 pN that cannot be detected by the HP-sensor module. Contrary to our expectation, the average force across Talin decreases during muscle compaction while tissue tension builds up and myofibrils are assembled.

To substantiate these findings, we compared flies carrying the HP-based Talin sensor (6–8 pN) to those with the stable variant HPst (9–11 pN), which only differs in 2 point mutations. We found similar and highly reproducible differences in FRET efficiency (Fig 5E, S4C Fig) indicating that, at 20 to 24 h APF, some Talin molecules even experience forces of ≥10 pN at muscle attachment sites. Comparison of TS to its stable variant (stTS) revealed a significant difference in FRET efficiency at 20 h APF, while the respective zero-force controls were indistinguishable (Fig 5E). This demonstrates that a proportion of the mechanically engaged Talin molecules experience a range of forces between 7 and 10 pN at muscle–tendon attachments in vivo, further emphasizing that the observed differences are force specific.

To test whether the remaining Talin molecules experience forces that are too low to be detected by the HP or HPst sensor modules, we generated flies with the F40 sensor module, which is sensitive to forces of 1 to 6 pN [13]. Again, we quantified a decrease in FRET efficiency relative to the control at 20 h and 24 h APF, but FRET efficiency differences remained small, and no change was observed at 30 h APF (Fig 5F). Thus, a large proportion of the Talin molecules at muscle attachment sites are not exposed to detectable mechanical forces during development.

To quantify the proportion of mechanically engaged Talin molecules at 20 h and 24 h APF, we applied biexponential fitting to our FLIM data and calculated the ratio of open versus closed sensor (Fig 5G, see Methods for details). This analysis revealed that only 13.2% and 9.6% of all Talin molecules are mechanically engaged at 20 h and 24 h APF, which contrasts in vitro measurements of focal adhesions that are characterized by a Talin engagement ratio of about 70% [12].

Each mechanically engaged Talin molecule needs to be bound to an integrin, therefore we tested whether the integrin levels at muscle attachment sites may be limiting the amount of force-bearing Talin molecules. However, integrins and Talin are present at comparable levels at muscle attachment sites at 20 h and 30 h APF (S5 Fig). Thus, it is unlikely that a lack of integrins is the primary reason for the surprisingly small proportion of Talin molecules experiencing detectable forces.

### Talin concentration at developing muscle attachments

**Fig 6:**
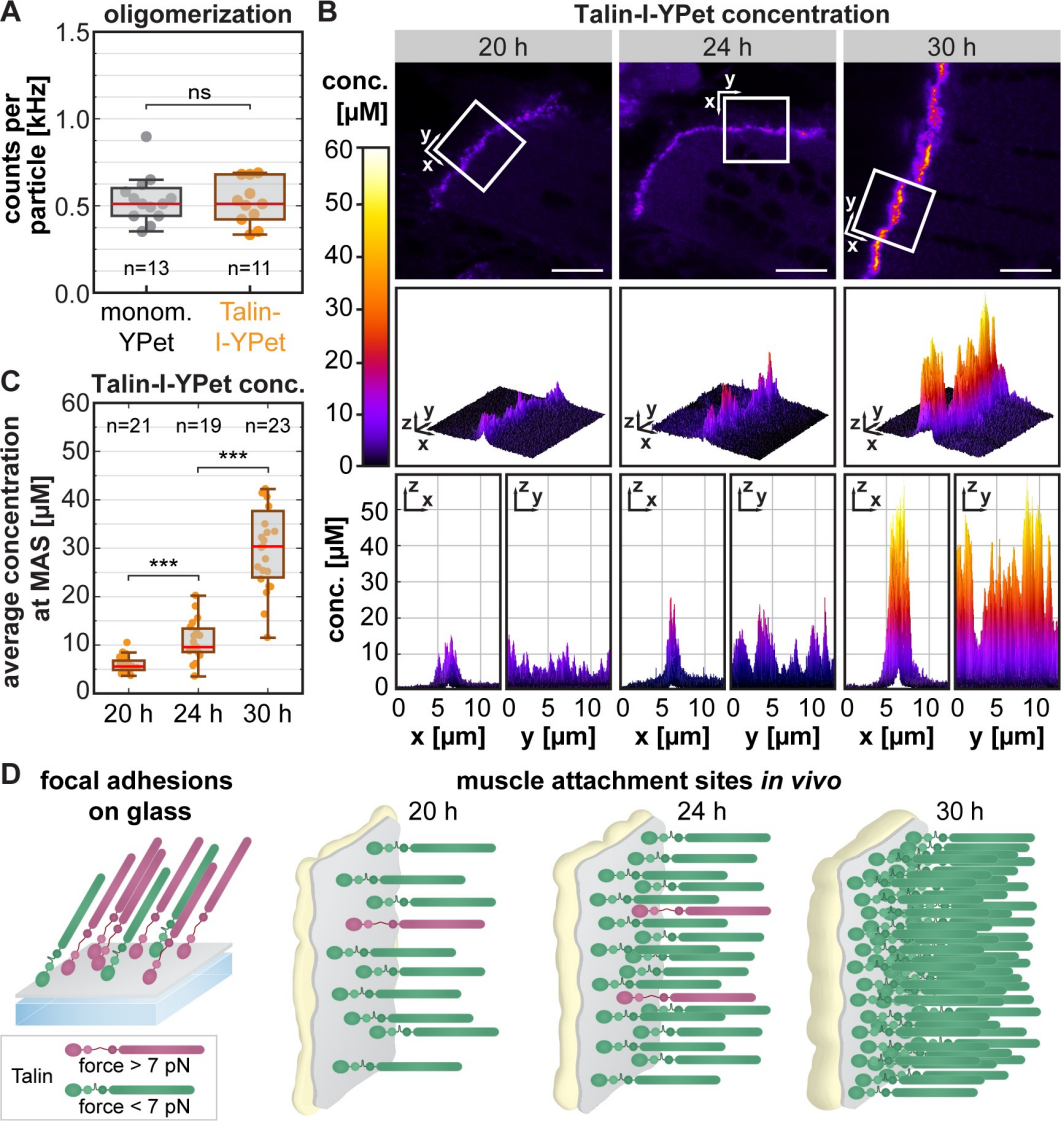
Talin concentration at muscle attachment sites increases 5-fold during attachment maturation. (A) Degree of Talin oligomerization measured by in vivo FCS in the muscle interior. Brightness (in CPP) of monomeric free YPet compared to Talin-I-YPet particles. Note that Talin-I-YPet particles are as bright as monomeric YPet, thus Talin-I-YPet is also monomeric (Kolmogorov-Smirnov test; ns: *p* > 0.05). (B) Absolute Talin-I-YPet concentration measured by FCS in combination with quantitative confocal imaging. Representative calibrated concentration images are shown for 20, 24, and 30 h APF. The boxes mark the area shown in the graphs below from different perspectives as indicated. Scale bars are 10 μm. (C) Quantification of the average Talin-I-YPet concentration at the MASs per image. Note that the concentration increases about 2-fold from 20 h to 24 h APF and 5-fold to 30 h APF. (Kolmogorov-Smirnov test, ***p < 0.001) (D) Model of mechanical Talin engagement. In focal adhesions, 70% of the Talin molecules are under force [12], whereas at developing MAS in vivo, less than 15% are mechanically engaged at any given time. As more Talin is recruited during muscle attachment maturation, the proportion of mechanically engaged Talin molecules decreases even further. Underlying data can be found in S1 Data. conc., concentration; CPP, counts per particle; FCS, fluorescence correlation spectroscopy; h APF, hours after puparium formation; I-YPet, Talin with internal YPet; MAS, muscle attachment site; ns, nonsignificant; YPet, yellow fluorescent protein for energy transfer.

As Talin is thought to play an important mechanical role during tissue formation, we wanted to test whether such a small proportion of mechanically engaged Talin molecules in vivo could still contribute a significant amount of tissue-level tension. We therefore quantified the absolute amount of Talin molecules present at muscle attachment sites by combining in vivo fluorescence correlation spectroscopy (FCS) with quantitative confocal imaging (see workflow in S6A–S6D Fig). From FCS measurements in the muscle interior, we calculated the counts per particle (CPP) value, i.e. the molecular brightness of a single Talin-I-YPet particle in each pupa. Because such a particle may correspond to a Talin monomer or dimer, we compared the Talin-I*-*YPet brightness to the brightness of free monomeric YPet expressed in flight muscles and found no significant difference (Fig 6A). We conclude that Talin is mostly monomeric in the muscle interior.

Next, we calculated the Talin concentration at muscle attachment sites by calibrating confocal images using the molecular brightness (CPP) information from the FCS measurements. Using a dilution series of Atto488, we ascertained that the fluorescence intensity increases linearly with the concentration over multiple orders of magnitude in our confocal images (S6E Fig). The resulting images with pixel-by-pixel Talin concentration values (Fig 6B) indicate an average concentration at the muscle attachment of 5.9 μM (20 h), 10.9 μM (24 h), and 30.9 μM (30 h) (Fig 6C). Thus, the local concentration of Talin molecules increases approximately 2-fold from 20 h to 24 h APF and 5-fold to 30 h APF, indicating that Talin may contribute to the high tissue stress by its strong recruitment to maturing muscle attachment sites.

To confirm this hypothesis, we estimated the density of Talin molecules on the membrane by dividing the number of Talin molecules per pixel by the estimated membrane area in the confocal volume (Fig 6D, see Methods for details). This resulted in about 400, 700, and 2,300 Talin molecules per μm^2^ at 20, 24, and 30 h APF, respectively, which corresponds to 20 nm × 20 nm space per molecule at 30 h APF. This space can easily accommodate the size of a Talin head domain (about 4 nm × 10 nm) [38], and the estimated density is comparable to previous studies of integrins in focal adhesions [39].

By combining our force quantifications with the estimated Talin density at muscle attachment sites, we calculated the Talin-mediated tissue stress to be in the order of 0.4 to 0.5 kPa at 20 h to 24 h APF (see Methods for details). These values are remarkably close to a previously published stress estimate of 0.16 kPa determined by traction force microscopy in focal adhesions of cultured cells [40]. Thus, Talin does contribute a significant amount of tissue stress despite the small proportion of mechanically engaged molecules (Fig 6D).

### High Talin levels are required to resist muscle contractions

To investigate the physiological relevance of the high Talin levels at muscle attachments, we aimed to reduce the Talin concentration. The simplest way would be to examine heterozygous animals with only 1 functional Talin copy. However, crossing *talin-I-YPet* to a *talin* null allele resulted only in a minor reduction of Talin levels (to about 80% of the WT level) at 20 h and 30 h APF muscle attachments (S7A–S7E Fig). Consequently, we did not detect any significant differences in the molecular forces across Talin in these heterozygous animals (S7F Fig).

**Fig 7:**
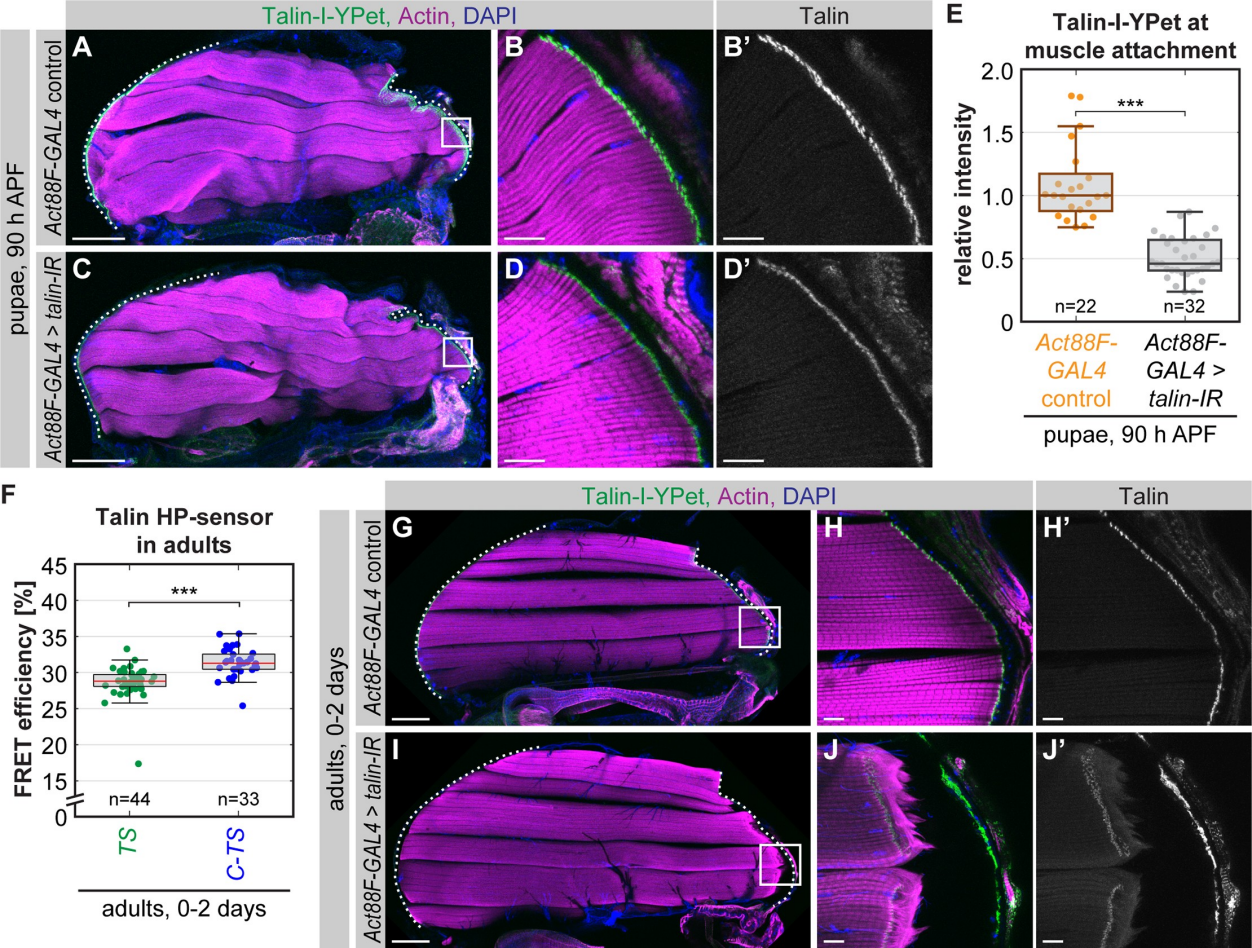
Reduced Talin levels lead to muscle attachment rupture in adults. (A–D) Talin knockdown in 90 h APF pupae. *Act88F*-GAL4 *;; talin-I-YPet* flies were crossed to *UAS-talin-IR* or WT flies as a control. Hemithoraxes of 90 h APF pupae were stained with phalloidin (actin) and DAPI. White boxes in A and C indicate zoom-in areas shown in B and D. The dotted lines highlight the cuticle. (E) Quantification of Talin-I-YPet intensity at muscle attachment sites. Median control intensity was set to 1. (Kolmogorov-Smirnov test, ***p < 0.001). (F) Talin forces in living whole-mount adults measured by FLIM-FRET. (Kolmogorov-Smirnov test, *** *p* < 0.001). (G–J) Talin knockdown phenotype in adults. Control and RNAi adult hemithoraxes were dissected and stained with phalloidin (actin) and DAPI. White boxes in G and I indicate zoom-in areas shown in H and J. The dotted lines highlight the cuticle. Note the ruptured muscle attachment in the *talin* knockdown condition. Scale bars are 100 μm in A, C, G, and I and 10 μm in B, D, H, and J. Underlying data can be found in S1 Data. C-TS, Talin control sensor with HP-sensor module; FLIM, fluorescence lifetime imaging microscopy; FRET, Förster resonance energy transfer; h APF, hours after puparium formation; HP, Villin headpiece; I-YPet Talin with internal YPet; *talin-IR*, *talin* RNA interference; TS, Talin tension sensor with HP-sensor module; WT, wild-type; YPet, yellow fluorescent protein for energy transfer.

Hence, we applied RNA interference (RNAi) to reduce Talin levels. As knockdown of Talin with a general muscle GAL4 driver—such as *Mef2*-GAL4—is embryonic lethal [30], we used the late flight-muscle–specific *Act88F*-GAL4 driver [41]. *Act88F*-GAL4–driven *talin* RNAi (*talin-IR*) resulted in a reduction of Talin levels to about 50% at flight muscle attachments at 90 h APF, which is shortly before the adult flies eclose (Fig 7A–7E). Apart from the reduced Talin levels, the muscle attachments look normal, and all flight muscles remain attached at 90 h APF.

As flight muscles at 90 h APF display a wavy shape and their cuticle has not hardened yet, we instead performed force measurements in adult flies, which have straightened flight muscles and are ready to fly. Talin force measurements in adult flight muscle attachments revealed a significant reduction in FRET efficiency for Talin-TS compared to the zero-force control. This indicates that a proportion of the Talin molecules indeed experience forces above 6 to 8 pN in adults under resting nonflying conditions (Fig 7F, S8 Fig) and the additional Talin could buffer peak muscle forces during flight.

To test this hypothesis, we investigated whether the reduction of Talin levels by RNAi has consequences during adult stages when flies actively fly and thus produce very high forces on muscle attachments. Indeed, *talin* knockdown flies display a muscle detachment phenotype, whereas in control flies, all muscles remain attached (Fig 7G–7J). In conclusion, high Talin levels are required for stable muscle attachments that withstand the high forces generated by active muscle contractions in adult animals.

## Discussion

Our findings highlight the importance of investigating tissues in their natural mechanical environment in vivo. While the forces per Talin molecule and the tissue stress in vivo are in the same order of magnitude as in previous in vitro studies of focal adhesions [11,12,40], a surprisingly small proportion of Talin molecules (<15%) experience detectable forces during muscle development in vivo. An obvious question arising, therefore, is: what are the other Talin molecules doing at muscle attachment sites, for which we cannot detect significant mechanical forces? Likely, the pool of mechanically engaged Talin molecules exchanges dynamically with the other Talin molecules present at the muscle attachment site. Talin molecules may even remain anchored to integrin and actin, without actomyosin pulling on them continuously. Such a dynamic system would allow the rapid adjustment to changes in tissue forces and thereby prevent rupture of the muscle–tendon attachment upon a sudden increase in tissue stress. In line with this hypothesis, we demonstrated that a high Talin level is particularly important when active muscle contractions result in high forces on the attachments.

Talin was just recently proposed to act as a “shock absorber” based on cell culture experiments [26]. In focal adhesions of cultured cells, the length of Talin can fluctuate dynamically on the time scale of seconds, with Talin being transiently extended from 50 nm up to 350 nm [42]. This can be explained by reversible folding and unfolding of some of the 13 helical bundles in the Talin rod upon actomyosin-dependent stretching of Talin. The hypothesis that Talin acts as a shock absorber is consistent with our finding that only some molecules experience forces at the same time under baseline conditions, whereas additional molecules may dampen a force increase. A similar force-induced reversible unfolding mechanism was recently proposed for particular immunoglobulin domains in the giant sarcomeric protein titin during muscle contraction cycles at estimated forces of 6 to 8 pN [43]. Thus, it is conceivable that muscle attachments prepare for peak forces during muscle contraction cycles by the recruitment of large amounts of Talin during development.

In addition, the unfolding of the Talin rod domains makes binding sites accessible, leading to the recruitment of vinculin [44]. Magnetic tweezer-based in vitro studies suggested that the rod domain R3 unfolds at about 5 pN [45] and the remaining rod domains unfold when forces larger than 8 pN are applied [46]. Our in vivo force measurements are consistent with those observations suggesting that low pN forces change the Talin structure and make vinculin binding sites accessible, thereby allowing a mechanotransduction response.

Previous estimates of forces transmitted by integrins based on studies of focal adhesions in vitro cover a wide range of forces. Studies using extracellular sensors with synthetic integrin ligands (that report forces based on double-stranded DNA rupture) suggest that integrins can experience very high forces in cells plated on glass (more than 54 pN) [47,48]. However, other data generated with FRET-based extracellular sensors suggest that about 70% of the integrins in focal adhesions experience low forces (less than 3 pN) [49]. These in vitro systems have the advantage that they are accessible for precise manipulations; however, the artificial mechanical environment may have a strong impact on the amount of force experienced by the individual proteins and the number of molecules that are mechanically engaged. Our study provides, to our knowledge, the first insights into molecular forces acting on integrin-mediated attachments in vivo. Here, we focused on developing muscle attachments in pupae; however, our newly established Talin tension sensor fly lines should enable future force measurements in all integrin-based processes in *Drosophila* leading to more insights into mechanobiology in vivo.

In this study, we found that only a small proportion of Talin molecules (<15%) are experiencing forces higher than 6 to 8 pN at developing muscle attachments and thus hypothesize that tissues prevent mechanical failure in vivo with the following mechanism: a large pool of molecules dynamically share the mechanical load, such that a sudden increase in tissue tension can be rapidly buffered by mechanically engaging additional molecules already present at the attachment site. These additional molecules could either be unbound and then rapidly recruited or already bound but not yet under force. Mechanical failure of integrin-mediated attachments in vivo needs to be avoided at all cost, particularly in muscle fibers or cardiomyocytes, to prevent fatal consequences for the animal. Therefore, creating a mechanical buffer system to withstand peak forces is an important concept for the survival of animals.

## Methods

### Fly strains

All fly work was performed at 27°C to be consistent with previously published work, unless otherwise stated. For details on the genome engineering strategy resulting in Talin tension sensor and control stocks generated in this study (*talin-F40-TS*, *talin-C-F40-TS*, *talin-TS*, *talin-C-TS*, *talin-stTS*, *talin-C-stTS*, *talin-I-YPet*, *talin-C-YPet*, *talin-I-mCh*, and *talin-C-mCh*), see below. Muscles were labelled using *Mef2*-GAL4 [50] with *UAS*-mCherry-Gma [51]. To label the tendon and muscle tissue simultaneously, *Mef2*-GAL4 and *stripe*-GAL4 [52] were used in combination with *UAS*-brainbow [53]. For quantifying Talin-GFP levels, a MiMIC GFP-trap line was used [54]; for Integrin-GFP levels, a homozygous viable GFP knockin line was used [55]. The deficiency line *rhea*^*79*^ was used as a *talin* null allele [31]; to achieve Talin knockdown, *Act88F*-GAL4 [41] was crossed to *UAS-talin-IR* (TF40399, obtained from the VDRC stock center [56]) at 25°C.

### Generation of tension sensor and control stocks

Tension sensor and control stocks were generated by combining CRISPR/Cas9-mediated genome engineering with ϕC31-mediated cassette exchange as described previously [28]. See S1 Fig for a detailed depiction of the two-step strategy. For step 1, single guide RNAs (sgRNAs) were designed with the help of an online tool maintained by the Feng Zhang lab (http://crispr.mit.edu/) [57] and transcribed in vitro. After testing sgRNA cutting efficiency in Cas9-expressing S2-cells [58], 2 sgRNAs (70 ng/μL) were injected into *Act5C-Cas9*, *DNAlig4*^*169*^ embryos together with the dsRed donor vector (500 ng/μL) containing a dsRed eye marker cassette flanked by attP sites and homology arms. Successful homologous recombination events were identified by screening for red fluorescent eyes and verified by PCR and sequencing. “Ends-in” events were excluded. We call the resulting fly lines *talin-I-dsRed* and *talin-C-dsRed*. For step 2, vasa-ϕC31 plasmid (200 ng/μL) was injected together with attB-donor vector (150 ng/μL). Successful exchange events were identified by screening for the absence of dsRed, and correct orientation of the cassette was verified by PCR.

### Adult hemithorax staining

Adult hemithoraxes were dissected and stained similar to as previously described [59]. Specifically, the wings and abdomen were cut off the thorax of adult flies with fine scissors, and the thoraxes were fixed for 15 min in 4% PFA in relaxing solution (20 mM sodium phosphate buffer [pH 7.0], 5 mM MgCl_2_, 5 mM ATP, 5 mM EGTA, 0.3% Trition-X-100). After washing once with PBST (PBS with 0.3% Triton-X-100), the thoraxes were placed on double-sided tape, and the legs were cut off. Next, the thoraxes were cut sagittally with a microtome blade (dorsal to ventral). The thorax halves were placed in PBST, washed once, and blocked in normal goat serum (1:30) for 30 min at room temperature (RT) on a shaker. Primary antibodies (anti-Talin antibody: 1:500, 1:1 mixture of E16B and A22A, DSHB) were incubated overnight at 4°C on a shaker. Hemithoraxes were then washed 3 times 10 min in PBST at RT and stained with secondary antibody (Alexa488 goat antimouse IgG, 1:500, Molecular Probes) and phalloidin (Rhodamine or Alexa647 conjugate, 1:500 or 1:200, respectively, Molecular Probes) in PBST for 2 h at RT in the dark. After washing 3 times with PBST for 5 min, hemithoraxes were mounted in Vectashield containing DAPI with 2 spacer coverslips on each side. YPet signal after fixation was bright enough for imaging without further amplification.

### Dissection of pupae

At 32 h APF, pupae were freed from the pupal case and dissected in PBS in a silicone dish using insect pins [59]. The head and the sides were cut using fine scissors to remove the ventral half of the pupa. Next, the thorax was cut sagittally, and the thorax halves were cut off the abdomen and placed in fixing solution (4% PFA in PBST) for 15 min. The thorax halves were then stained with phalloidin and DAPI like the adult hemithoraxes but without shaking and were mounted using 1 spacer coverslip. At 90 h APF, pupae were dissected like adults after freeing them from the pupal case (see above).

### Imaging of stainings

Samples were imaged on a Zeiss LSM 780 scanning confocal microscope with Plan Apochromat objectives (10× air, NA 0.45 for overview images and 40× oil, NA 1.4 for detail images). For thick samples, a z-stack was acquired and maximum-projected using the ImageJ variant Fiji [60].

### Sarcomere length quantification

Sarcomere length was quantified as previously described using the Fiji plug-in MyofibrilJ (https://imagej.net/MyofibrilJ) [29]. Briefly, an area with straight, horizontal myofibrils is analyzed by Fourier transformation to find the periodicity of the sarcomeres. One area was analyzed for each hemithorax stained with phalloidin and imaged at 40× and zoom 4.

### Western blotting

Western blotting was performed according to standard procedures. Specifically, 15 flies each were homogenized in 100 μL 6× SDS loading buffer (250 mM Tris [pH 6.8], 30% glycerol, 1% SDS, 500 mM DTT) and heated to 95°C for 5 min. The amount of 200 μL water was added, and the equivalent of 0.5 fly (10 μL) and 1 fly (20 μL), respectively, were loaded onto a NuPAGE Novex 3–8% Tris-Acetate Gel. The transfer to the membrane was carried out overnight with 20 V at 4°C. The membrane was blocked (5% blotting grade blocker, BioRad) and then incubated overnight at 4°C with a 1:1 mixture of anti-Talin antibodies E16B and A22A (1:1,000 in block). For detection, HRP antimouse antibody and Immobilon Western Chemiluminescent HRP Substrate (Millipore) were used.

### Flight assays

Male flies (1–3 d old, aged at 25°C) were thrown into a 1 m × 8 cm plexiglass cylinder with 5 marked sections [56]. Flightless flies fall to the bottom of the tube immediately, whereas strong fliers land in the top 2 sections and weak fliers in the third and fourth section. Flight assays were performed in triplicates with 10–20 males each and were repeated twice.

### Live imaging of embryos and larvae

Embryos from the cross *yw; talin-I-YPet* to *w; Mef2*-GAL4 *; UAS*-mCherry-Gma were collected on apple juice agar plates for 24 h and dechorionated in 50% bleach (0.024% hypochlorite) for 3 min. Living embryos were mounted in 50% glycerol before imaging. L3 larvae from the same cross were immobilized by immersing them in 60°C water for about 1 s [30] and mounted using a plexiglass slide with a groove and 1 spacer coverslip on each side in 50% glycerol. Five-by-1–tile scan z-stacks were acquired using a 10× objective to image the entire larva.

### Sample preparation for live imaging of pupae and adult flies

White pre-pupae were collected and aged at 27°C to the desired time point. Before imaging, a window was cut into the pupal case above the thorax, and the pupae were mounted on a custom-made slide with a groove as previously described [61].

Living adults (0–2 d after eclosion) were mounted similarly: after cutting off the legs to prevent the flies from moving too much, up to 5 flies were each placed in a small drop of 50% glycerol (with 0.13% Triton to ensure that the fluid can wet the water-repellent surface of the cuticle) on a coverslip on their dorsal sides. The wings were then spread out in the drops on the coverslip, and the flies were aligned in a row anterior to posterior. Next, the coverslip was flipped over and placed on a custom-made slide with a groove and 2 spacer coverslips, such that the groove accommodated all 5 flies. In this way, the anterior muscle attachment sites of the dorsal most flight muscles can be imaged directly through the adult cuticle. The flies on each slide were imaged immediately after mounting to minimize the amount of time that they had spent confined to the slide before the measurement.

### FRAP

Living 24 h APF *talin-C-YPet* or *talin-TS* pupae were imaged at 25°C on a Leica SP8 scanning confocal microscope equipped with an argon laser. A 63× water objective (HC PL APO CS, NA 1.2) was used at zoom 2 to image flight muscle attachment sites first for 5 frames before the bleach (512 × 512 px), then a region of interest (ROI; 120 × 40 px) was bleached for 1 frame using all 4 argon laser lines (458 nm, 476 nm, 488 nm, and 514 nm), and finally the fluorescence recovery was followed for 61 frames with a 5 s time resolution. The resulting 5-min movies were analyzed with the Fiji plug-in FRAP profiler (http://worms.zoology.wisc.edu/ImageJ/FRAP_Profiler.java) by comparing the bleached region to a control region of the muscle attachment to correct for gradual bleaching during image acquisition. FRAP curves were each normalized (1 = pre-bleach intensity; 0 = intensity directly after bleaching) and then fit with a single exponential, yielding the recovery half time and the mobile fraction. Movies in which the attachment moved out of plane or out of the bleached region were excluded from the analysis. The experiment was performed on 3 independent experiment days.

### Isolation and differentiation of primary muscle fibers

Primary cells were isolated from *Drosophila* embryos and differentiated as previously described [33,34] with the following modifications: embryos (5–7 h old, aged at 25°C) were collected from smaller cages on only one 9-cm molasses plate per genotype. Embryos were homogenized with a Dounce homogenizer using a loose-fit pestle in 4 mL Schneider’s *Drosophila* medium (Gibco 21720–024, lot 1668085) and, after several washing steps (using 2 mL medium), were resuspended to a concentration of 3 × 10^6^ cells/mL. Finally, cells were plated in 8-well ibidi dishes (1 cm^2^ plastic bottom for microscopy with ibiTreat surface) coated with vitronectin (optional) at a density of 3–9 × 10^5^ cells/cm^2^ and differentiated for 5 to 7 d at 25°C in a humid chamber.

### Fixation, staining, and imaging of primary muscle fibers

Primary muscle fibers were fixed on day 6 after isolation with 4% PFA in PBS for 10 min at RT on a shaker. Phalloidin-staining (Alexa647-conjugate; Molecular Probes) was performed overnight in the dark at 4°C. Fixed cells were imaged in PBS on a Zeiss LSM 780 with a 40× oil objective (Plan Apochromat, NA 1.4). Live imaging of twitching primary cells was performed on a Leica SP5 confocal with a 63× water objective (HCX PL APO 63×/1.2 W CORR λ_BL_), acquiring the transmission light channel and the YPet channel simultaneously.

### Tissue tension analysis by laser cutting

Laser cutting and imaging was performed similar to a previous study on a custom-built setup with a spinning disc unit and a UV laser (355 nm, 100 mW nominal power) [37]. Here, flight muscles and the connected tendon tissue were imaged at 20 h and 30 h APF in *stripe*-GAL4, *Mef2*-GAL4, *UAS*-brainbow pupae expressing palmitoylated mCherry as a marker in the tendon and muscle tissue or in *talin-I-YPet* pupae with Talin-I-YPet as marker for muscle attachment sites. For performing line cuts in a single z-plane, movies were acquired with a 300-ms time resolution for 150 frames (45 s) using a 40× water objective (NA 1.1, Leica). After the first 10 frames, an 80- to 100-μm–long line was cut (UV laser pulse repetition rate: 1 kHz, 2 pulses every 0.5 μm) into the tendon or muscle tissue, and the recoil was followed over time. For performing line cuts in a 10-μm–thick z-stack in the muscle fibers, z-stack movies were acquired with a z-spacing of 1 μm and an exposure time of 300 ms per slice, resulting in 5.3 s acquisition time per stack. A total of 10 frames were acquired (42.5 s). During the second frame, 5 line cuts were performed, thereby cutting the tissue every 2 μm in z. A single z-plane of the resulting movie was chosen to analyze the tissue recoil.

To quantify the tissue recoil, a line (20 px width) was drawn along the direction of the movement in Fiji, and a kymograph with the average intensity along the line over time was created using the plug-in KymographBuilder [62]. In the kymograph, the movement of the tendon tissue or the muscle attachment was tracked manually by using the multipoint tool and the measure function. The initial recoil velocity of the tendon tissue was calculated from the first 2 frames after the cut. The recoil velocity of the muscle attachment after cutting the muscle in a z-stack was calculated from the position of the attachment in the first frame after the cut (at 5.3 s) compared to the position before the cut.

### FLIM

Primary muscle fibers and pupae were imaged live on a Leica SP5 microscope equipped with a pulsed white light laser (NKT Photonics, 80 MHz), a time-correlated single photon counting (TCSPC)-FLIM detector (FLIM X16, LaVision BioTec), and a 545/30 nm emission filter (Chroma). Primary muscle fibers were imaged with a 63× water objective (HCX PL APO 63×/1.2 W CORR λ_BL_), and pupae were imaged with a 40× water objective (HC PL APO 40×/1.1 W CORR CS2). Photon arrival times were detected with a resolution of 0.08 ns in a 12.5 ns time window between laser pulses.

### FLIM-FRET data analysis

The FLIM data were analyzed using a custom-written MATLAB (MathWorks) program [11,12]. First, an intensity image was created to manually draw an ROI around the target structure (adhesions/costameres in primary cells or muscle attachment sites in pupae, also see S3 Fig). To create a binary mask of the target structure, Multi-Otsu thresholding with 3 classes was applied to the signal in the ROI blurred with a median filter (3 × 3 pixels), and holes in the mask containing the brightest class were filled. Photon arrival times of all photons inside the mask were plotted in a histogram, and the tail of the curve was fitted with a monoexponential decay yielding the fluorescence lifetime *τ*. Fits with more than 5% relative error in lifetime determination were excluded from further analysis. For dimmer samples (primary fiber cultures and intermolecular FRET pupae), we used a 10% relative error cut-off. The FRET efficiency *E* was calculated according to the following formula, with *τ*_*DA*_ being the lifetime of the donor in presence of the acceptor and *τ*_*D*_ the lifetime of the donor alone:

$$E=1-\frac{\tau_{DA}}{\tau_{D}}$$ (1)

For all measurements, *τ*_*D*_ was determined as the median lifetime of Talin-I-YPet in the same experimental conditions. Experiments were repeated 2 to 5 times on different experiment days with 10 to 15 pupae/cells imaged per genotype and day.

### Calculation of the proportion of mechanically engaged Talin

We determined the number of mechanically engaged (= open) tension sensor *N*_*open*_ relative to the total number of molecules *N*_*total*_ at the muscle attachment site using biexponential fitting similar to as previously described [12]. Briefly, we assumed that the fluorescence decay from a tension sensor FLIM measurement can be described by 2 lifetimes: the lifetime of the open sensor *τ*_*noFRET*_ and the lifetime of the closed sensor undergoing FRET *τ*_*FRET*_. The lifetime of the open sensor *τ*_*noFRET*_ approximately corresponds to the lifetime of the donor alone, because of the large contour length increase upon opening of the sensor. Thus, we determined the lifetime *τ*_*noFRET*_ by using a monoexponential fit on Talin-I-YPet data as described above. The lifetime *τ*_*FRET*_ was determined from zero-force control (Talin-C-TS) data. Since the Talin-C-TS sample contains fully fluorescent sensor (*τ*_*FRET*_) and sensor with nonfluorescent mCherry acceptor (*τ*_*noFRET*_), we used a biexponential fit with fixed *τ*_*noFRET*_ to determine *τ*_*FRET*_. The 2 lifetimes *τ*_*noFRET*_ and *τ*_*FRET*_ were then fixed and used to fit Talin-TS and Talin-C-TS data biexponentially, thereby determining the relative contributions of photons from molecules with these two lifetimes. From this, the relative number of molecules with *τ*_*noFRET*_ and *τ*_*FRET*_ was calculated, taking into account that FRET reduces the number of photons detected in the donor channel. Finally, the ratio *N*_*open*_*/N*_*total*_ was determined by normalizing the Talin-TS values to the respective Talin-C-TS values.

### Relative quantification of protein levels

For the relative quantification of Talin-GFP and Integrin-GFP (βPS-GFP, Mys-GFP) levels at flight muscle attachments, living 20 h and 30 h APF pupae (mounted as described above) were imaged on a Zeiss LSM 780 scanning confocal microscope with a 40× oil objective (Plan Apochromat, NA 1.4) using the same laser power and gain settings for Talin- and Integrin-GFP pupae. Muscle attachments were traced manually with the free-hand selection tool in Fiji using a fixed line width (40 px for 20 h APF and 20 px for 30 h APF). The intensity in the area along the line was averaged for each pupa. For each experiment day, the median Talin-GFP intensity of all pupae was set to 1, and the relative Integrin-GFP intensity was calculated. Finally, the data from 3 independent experiment days were merged. Because the Talin-GFP allele is not homozygous viable, both the Talin-GFP and the Integrin-GFP flies were crossed to WT flies for this experiment.

For quantifying Talin-I-YPet levels in heterozygous pupae, *talin-I-YPet* flies were crossed to *talin* null flies (deficiency *rhea*^*79*^) [31], and homozygous *talin-I-YPet* animals were used as a control. In addition to images acquired on the Zeiss LSM 780 microscope as described above, confocal images from the corresponding FLIM data set (S7F Fig) were used for quantification.

For quantifying Talin-I-YPet levels at muscle attachments at 90 h APF upon *talin* knockdown with *Act88F-*GAL4, z-stacks were acquired on the Zeiss LSM 780 microscope with a 10× air objective (Plan Apochromat objectives, NA 0.45). In a maximum-projected image of the thorax, anterior and posterior flight muscle attachments were traced manually with the free-hand selection tool in Fiji using a line width of 4 px. For this experiment, the flies were crossed at 25°C because at 27°C *Act88F*-GAL4 is detrimental.

### FCS

Living *talin-I-YPet* pupae were analyzed at 20, 24, and 30 h APF by a combination of confocal microscopy (LSM 780, Zeiss) and FCS using a 40× water objective (C-Apochromat 40×/1.20 W Korr UV-VIS-IR) and the built-in GaAsP detector in single photon counting mode. Prior to the experiment, the correction collar and pinhole position were adjusted with fluorescent Rhodamine 6G in aqueous solution (30 nM in Tris [pH 8]) using the same type of cover glass (Marienfeld, High Precision, 18 × 18 mm, 170 ± 5 μm thickness) as for mounting the pupae [61]. To calibrate the detection volume (excitation 514 nm laser light), we measured FCS (120 s recordings) at 3 different positions 20 μm above the cover glass surface. Autocorrelation curves were analyzed with our open-source software *PyCorrFit* [63] (version 1.0.1, available online at http://pycorrfit.craban.de/). For fitting Rhodamine 6G data, we used a model accounting for triplet transitions and three-dimensional diffusion (denoted “T-3D” in *PyCorrFit*). The detection volume *V*_*eff*_ was calculated based on the measured diffusion time (*τ*_*diff*_) and the published diffusion coefficient D = 414 μm^2^/s [64]:

$$V_{\mathrm{eff}}=S\cdot {\left(4\pi \cdot D\cdot \tau_{\mathrm{diff}}\right)}^{3/2}$$ (2)

For all measurements, the axis ratio of the detection volume S = 5 was consistently fixed [65].

In living pupae, fluorescent proteins (YPet or Talin-I-YPet) were measured by FCS using a park and probe procedure [66]: in images, 3 positions in the muscle interior next to the muscle attachment site were manually selected for FCS (10× 40 s recordings). For fitting of Talin-I-YPet autocorrelation curves (time bins > 1 μs), a two-component three-dimensional diffusion model with 2 nonfluorescent dark states (denoted “T+T+3D+3D” in *PyCorrFit*) was applied. Transient dark states were assigned either to triplet transitions (τ_trip1_, T_1_) in the time range of 1–20 μs and photochemical flickering (τ_trip2_, T_2_) in the time range of about 200–600 μs [67]. The first diffusion time was assigned to protein diffusion in the muscle interior, whereas the second diffusion term was merely a descriptive term accounting for slow long-tail behaviour that cannot be avoided in a crowed intracellular environment [66]. Autocorrelation curves derived from visibly unstable intensity traces were excluded from further analysis. Due to the high endogenous expression levels, the contribution of noncorrelated background was negligible. Thus, the molecular brightness, i.e., the CPP value, of Talin-I-YPet was determined by dividing the average intensity *I* (brackets indicate the average) by the number of molecules in the focal volume *N*, which is dependent on the autocorrelation amplitude G(0) of the autocorrelation function G(τ) and the dark fractions T_1_ and T_2_ from the fit:

$$CPP=\frac{\langle I\rangle }{N}=\langle I\rangle \cdot G\left(0\right)\cdot \left(1-T_{1}-T_{2}\right)$$ (3)

Because freely diffusing YPet diffuses faster than Talin-I-YPet, the signal fluctuations related to flickering and diffusion cannot be distinguished in YPet measurements. Therefore, the autocorrelation curves of free YPet were fitted by a simplified model function accounting only for transient triplet states and 2 diffusive terms, of which the first combines contributions of both protein diffusion and flickering (denoted “T-3D-3D” in *PyCorrFit*). To estimate true particle numbers, we corrected for triplet transitions and flickering globally by using the average fractions T_1_ and T_2_ from corresponding Talin-I-YPet measurements performed with the same excitation power density:

$$CPP_{YPet}=\frac{\langle I\rangle }{\langle N\rangle }=\langle I\rangle \cdot \langle G(0)\rangle \cdot \left(1-\langle T_{1,Talin‐I‐YPet}\rangle -\langle T_{2,Talin‐I‐YPet}\rangle \right)$$ (4)

The diffusion constant of freely expressed YPet was in good agreement to other fluorescent proteins in the cytoplasm of living cells, suggesting that the point spread function positioned in the muscle cell is still diffraction limited. This finding justifies the external calibration of the detection volume by Rhodamine 6G.

### Calibration of confocal images

For quantification of the absolute Talin-I-YPet concentration at muscle–tendon attachment sites, the developing flight muscles were imaged in photon counting mode (512 × 512 px, pixel dwell time *PT* = 50 μs). Saturation of the detector was carefully avoided by keeping *I*(x,y) below 2 MHz. The counts in each pixel of an image were calibrated by the molecular brightness (CPP) value determined for Talin-I-YPet in the interior of the same muscle fiber by FCS [66]. Due to the monomeric state of Talin-I-YPet, intensity values stored in each pixel *I*(x,*y*) could be directly transformed into numbers of Talin molecules:

$$N\left(x,y\right)=\frac{I\left(x,y\right)}{CPP\cdot PT}$$ (5)

Using the Avogadro constant (*N*_*A*_) and the detection volume (*V*_*eff*_) as determined by Rhodamine 6G measurements, we then calculated concentration maps:

$$c\left(x,y\right)=\frac{N\left(x,y\right)}{N_{A}\cdot V_{\mathrm{eff}}}$$ (6)

Finally, the muscle attachment sites were isolated in the Talin-I-YPet concentration maps by creating a mask with the same thresholding algorithm as used for FLIM-FRET. The concentration values were averaged across pixels within the mask resulting in a mean concentration value per pupa.

A prerequisite for this approach is that the count values per pixel in the acquired confocal images increase linearly with the concentration of the analyte. To test this, we made an Atto488 1:10 dilution series and acquired confocal images 50 μm into a drop of each dilution on a coverslip (covered to prevent evaporation). Quantification of the images indeed revealed a linear relationship between the photon count values and the analyte concentration over 5 orders of magnitude. Thus, low photon count values from Talin-I-YPet in the muscle interior can be directly compared to the high photon count values at the muscle attachment sites.

### Estimation of Talin density and tissue stress

To estimate Talin density on the membrane from pixel-by-pixel concentration values, we divided the average number of molecules in the focal volume at the muscle attachment sites by the membrane area in the focal volume. The focal volume was determined by Rhodamine 6G FCS measurements as described above. For the shape of the focal volume, we assumed an ellipsoid with the long axis (z) being 5 times the short axis (x = y). Therefore, for a focus volume of 0.32 fL, the membrane area in the z-y plane is 0.63 μm^2^. Taking into account that there are 2 membranes (one from the tendon and one from the muscle) and that the membrane is not flat (ruffles approximately increase the area 2-fold as determined from EM images [68]), the total membrane area in the focal volume is about 2.5 μm^2^.

To estimate Talin-mediated tissue stress, we calculated *force threshold of sensor* × *Talin density* × *proportion of mechanically engaged Talin* = 7 pN × 400 molecules/μm^2^ × 13.2% = 0.37 kPa for 20 h APF; and 7 pN × 700 molecules/μm^2^ × 9.6% = 0.47 kPa for 24 h APF. Note that these values are lower estimates because individual molecules might experience forces higher than 7 pN.

### Statistics

Box plots display the median as a horizontal line, and the box denotes the interquartile range. Whiskers extend to 1.5 times the interquartile range from the median and are shortened to the adjacent data point (Tukey). In addition, all data points are shown as dots. Tests used for statistical evaluation are indicated in the figure legends. All data and statistical tests are listed in S1 Data.

### Code availability

FLIM-FRET data were analyzed using custom-written MATLAB (MathWorks) code as published previously [11,12]. The code is available upon request.

## Supporting information

S1 Fig Talin tension sensor genome engineering. (A) Top: Gene model of *talin* (*rhea*, *isoform RF*) with the insertion sites (green) in the linker region between Talin head and rod (internal) and at the C-terminus. The gene *CG6638* immediately follows *talin* and therefore was also included. Middle: Tension sensor allele with the sensor module inserted into the target exon in the linker region of Talin. attR sites left in the surrounding introns are shown in light blue. Bottom: C-terminal control sensor allele with the sensor module inserted at the C-terminus of Talin. Gene models are drawn to scale. (B) Scheme showing how tension sensor alleles were generated. Step 1: The target exon in the linker region (green) was replaced by a splice acceptor (SA)-3×Stop-SV40 terminator (pA)-3×P3>dsRed-pA cassette flanked by attP sites (P) using the CRISPR/Cas9 system. Specifically, a dsRed donor vector containing 1.5 to 2.0 kb homology arms was injected into *Act5C*-Cas9 expressing embryos (also carrying a *DNAlig4*^*169*^ mutation to favor homology-directed repair over nonhomologous end-joining [28]) together with 2 in vitro*–*transcribed sgRNAs (target sites in blue). Successful targeting was identified by screening for fluorescent red eyes. Step 2: ϕC31-mediated cassette exchange was performed to replace the dsRed cassette by the original target exon including a tension sensor module consisting of YPet, a flexible, calibrated, mechanosensitive linker peptide (dark blue), and mCherry (mCh). To this end, a tension sensor donor vector including flanking attB sites (B) was injected together with *vasa*-ϕC31 plasmid. Thereby, the tension sensor was inserted seamlessly into the gene (after Talin amino acid 456) except for 2 attR sites (R) in the flanking introns. Successful exchange events were identified by screening for the absence of fluorescent red eyes [28]. Control fly lines with 1 fluorophore and fly lines with different tension sensor modules were generated by repeating step 2 with different donor vectors. (C) Scheme showing how C-terminal zero-force sensor alleles were generated using the same strategy. However, at the C-terminus, 3 exons (green) were replaced by the dsRed cassette in the first step, because the last intron in *talin* is small and the gene *CG6638* follows immediately after *talin*. All 3 exons were put back in the second step together with the sensor module resulting in 1 attR site in a *talin* intron and 1 in an *CG6638* intron. Respective controls with the individual fluorophores were also generated. CRISPR/Cas9, clustered regularly interspaced short palindromic repeats/CRISPR-associated protein 9; mCh, mCherry; sgRNA, single guide RNA.
(TIF)

S2 Fig Laser cutting induces contractions in 30 h APF flight muscles. (A–B) Stills from movies of *talin-I-YPet* pupae before and after an incomplete cut of the muscle in a single z-plane. Black lines mark the position of the laser cuts, and white lines indicate areas analyzed in kymographs in C and D. (C–D) Kymographs showing muscle attachment movement. Dashed lines mark the position of the muscle attachments before the cut (at 0 s). Time resolution is 300 ms. Scale bars are 10 μm. See S4 and S5 Movies. h APF, hours after puparium formation.
(TIF)

S3 Fig FLIM workflow to determine FRET efficiencies. (A) Living *talin-TS* or control pupae were prepared for imaging by opening a window in the pupal case above the thorax containing the developing flight muscles (magenta) [61]. (B) FLIM was performed on a confocal microscope equipped with a pulsed laser (indicated by green peak) for exciting the donor fluorophore (YPet) and a TCSPC detector for recording photon arrival times (indicated by yellow dot). (C) A YPet intensity image created from the FLIM data was used to manually draw an ROI containing the anterior muscle attachments sites of the dorsal-longitudinal flight muscles close to the surface of the thorax. From this ROI, a mask for the muscle attachment sites was created by Multi-Otsu thresholding. (D) Photon arrival times of all photons inside the mask were plotted in a histogram. The tail of the curve was fitted by a monoexponential decay to determine the lifetime *τ*. By comparing the lifetime of the Talin tension sensor *τ*_DA_ with the lifetime of respective donor-only control *τ*_D_, the FRET efficiency *E* was calculated. (E) Interpretation of FRET results: a high FRET efficiency indicates mostly closed sensor modules and therefore low force; vice versa, a low FRET efficiency indicates mostly open sensor modules and therefore high force. FLIM, fluorescence lifetime imaging microscopy; FRET, Förster resonance energy transfer; ROI, region of interest; TCSPC, time-correlated single photon counting; YPet, yellow fluorescent protein for energy transfer.
(TIF)

S4 Fig Control measurements for Talin forces detected at muscle attachment sites in vivo. (A) Lifetime data of donor-only controls at the internal position of Talin (I-YPet) (B) Lifetime data for I-YPet, C-YPet, TS, and C-TS at 24 h APF for each pupa plotted against the average intensity inside its muscle attachment site mask. Red dotted line represents median lifetime value. No correlation between lifetime and intensity could be detected (Pearson correlation coefficient *r* with 95% confidence interval and *p*-values are indicated). (C) Reproducibility of FLIM-FRET measurements performed in different years: TS and its stable variant stTS show a reproducible decrease in FRET efficiency compared to the C-terminal zero-force controls C-TS and C-stTS at 20 h APF (Kolmogorov-Smirnov test, ***p < 0.001, **p < 0.01, ns: *p* > 0.05). Underlying data can be found in S1 Data. C-TS, Talin control sensor with HP-sensor module; C-stTS, Talin control sensor with HPst-sensor module; C-YPet, Talin with C-terminal YPet; FLIM, fluorescence lifetime imaging microscopy; FRET, Förster resonance energy transfer; h APF, hours after puparium formation; HP, Villin headpiece; HPst, stable Villin headpiece; I-YPet, Talin with internal YPet; ns, nonsignificant; stTS, Talin tension sensor with HPst-sensor module; TS, Talin tension sensor with HP-sensor module; YPet, yellow fluorescent protein for energy transfer.
(TIF)

S5 Fig Integrin levels are comparable to Talin levels at muscle attachment sites. (A–D) Live imaging of flight muscle attachment sites at 20 h and 30 h APF. Pupae expressing Talin-GFP (A and C) were compared to pupae expressing Integrin-GFP (βPS-GFP) (B and D). Yellow shaded areas in A’–D’ indicate attachment regions in which GFP intensity was quantified. Scale bars are 10 μm. (E) Quantification of Talin- and Integrin-GFP intensity at muscle attachment sites. Median Talin-GFP intensity was set to 1 for 20 h APF and 30 h APF. (Kolmogorov-Smirnov test, ***p < 0.001, **p < 0.01). Underlying data can be found in S1 Data. GFP, green fluorescent protein; h APF, hours after puparium formation.
(TIF)

S6 Fig Quantitative imaging workflow and control measurements for FCS. (A) Living *talin-I-YPet* pupae were prepared for quantitative imaging by opening a window in the pupal case above the thorax containing the developing flight muscles (magenta) [61]. (B) A confocal image and 3 FCS measurements were acquired using the same detector on a confocal microscope. (C) Autocorrelation curves from the FCS measurements were fit to obtain a CPP value for each pupa. (D) The CPP value was used to calibrate each image resulting in a pixel-by-pixel concentration image. This image was used to manually draw an ROI around the muscle attachment site. From this ROI, a muscle attachment mask was created automatically by Multi-Otsu thresholding. Finally, the average concentration at the attachment was calculated from the pixel values inside the mask for each pupa. (E) Pixel-by-pixel photon count values measured in confocal images of an Atto488 dye dilution series (mean with standard deviation). Note that the number of detected photons increases linearly with the concentration of the dye for the entire range measured. Thus, the high intensities at muscle attachment sites can be directly compared to low intensities in the muscle interior of the same confocal image. Underlying data can be found in S1 Data. CPP, counts per particle; FCS, fluorescence correlation spectroscopy; ROI, region of interest.
(TIF)

S7 Fig Talin levels at muscle attachment sites are only slightly reduced in heterozygous pupae. (A–D) Live imaging of flight muscle attachment sites of homozygous *talin-I-YPet* pupae (2 copies) and heterozygous *talin-I-YPet/rhea*^*79*^ pupae (1 copy). Scale bars are 10 μm. (E) Quantification of Talin-I-YPet (I-YPet) intensities at muscle attachment sites. Median intensity of homozygous pupae was set to 1 for 20 h and 30 h APF. Note that the Talin levels are only reduced to 80% and not 50% in heterozygous pupae. (F) Talin force measurements in homozygous and heterozygous tension sensor (TS) and zero-force control (C-TS) pupae. (Kolmogorov-Smirnov test, ***p < 0.001, *p < 0.05; ns: *p* > 0.05). Underlying data can be found in S1 Data. C-TS, Talin control sensor with HP-sensor module; h APF, hours after puparium formation; HP, Villin headpiece; I-YPet, Talin with internal YPet; ns, nonsignificant; *rhea*^*79*^, *talin* deficiency; TS, Talin tension sensor with HP-sensor module; YPet, yellow fluorescent protein for energy transfer.
(TIF)

S8 Fig Control measurements for Talin forces detected at muscle attachment sites in adult flies. (A) Lifetime data of donor-only control at the internal position of Talin (I-YPet) in adults. The measured lifetime is slightly lower than in pupae at 18–30 h APF (S4A Fig), likely due to the short background lifetime of the adult cuticle that is just above to the muscle attachment sites. (B) Intermolecular FRET control data comparing heterozygous I-YPet/I-mCh or C-YPet/C-mCh pupae to homozygous I-YPet or C-YPet pupae, respectively. Underlying data can be found in S1 Data. C-mCh, Talin with C-terminal mCherry; C-YPet, Talin with C-terminal YPet; FRET, Förster resonance energy transfer; h APF, hours after puparium formation; I-mCh, Talin with internal mCherry; I-YPet, Talin with internal YPet; YPet, yellow fluorescent protein for energy transfer.
(TIF)

S1 Movie Live imaging of primary muscle fibers. Movie of the twitching primary muscle fiber shown in Fig 3F. Talin-I-YPet signal (green) is overlaid with the transmission light channel (grey) acquired simultaneously. The length of the movie is 1 min with a time resolution of 1.29 s played at 10× speed. Scale bar is 10 μm. I-YPet, Talin with internal YPet; YPet, yellow fluorescent protein for energy transfer.
(AVI)

S2 Movie Tendon tissue laser cutting at 20 h APF. Movie of the *stripe-*GAL4, *Mef2-*GAL4, *UAS-*brainbow pupa shown in Fig 4B. The black line indicates the position of the cut. The length of the movie is 45 s with a time resolution of 300 ms played at 5× speed. Scale bar is 10 μm. h APF, hours after puparium formation.
(AVI)

S3 Movie Tendon tissue laser cutting at 30 h APF. Movie of the *stripe-*GAL4, *Mef2-*GAL4, *UAS-*brainbow pupa shown in Fig 4C. The black line indicates the position of the cut. The length of the movie is 45 s with a time resolution of 300 ms played at 5× speed. Scale bar is 10 μm. h APF, hours after puparium formation.
(AVI)

S4 Movie Muscle laser cutting in a single z-plane at 20 h APF. Movie of the *talin-I-YPet* pupa shown in S2A Fig. The black line indicates the position of the cut. Note that there is no contraction of the muscle induced after the cut. The length of the movie is 45 s with a time resolution of 300 ms played at 5× speed. Two consecutive frames each were averaged to reduce file size. Scale bar is 10 μm. h APF, hours after puparium formation.
(AVI)

S5 Movie Muscle laser cutting in a single z-plane at 30 h APF. Movie of the *talin-I-YPet* pupa shown in S2B Fig. The black line indicates the position of the cut. Note that the muscle contracts after the cut, showing that the immature myofibrils at this stage are already contractile. The length of the movie is 45 s with a time resolution of 300 ms played at 5× speed. Two consecutive frames each were averaged to reduce file size. Scale bar is 10 μm. h APF, hours after puparium formation.
(AVI)

S6 Movie Muscle laser cutting in a z-stack at 20 h APF. Movie of the *talin-I-YPet* pupa shown in Fig 4H. The black line indicates the position of the z-stack cut. Note that the muscle attachment recoils away from the cut. The length of the movie is 42.5 s with a time resolution of 5.3 s played at 20× speed. Scale bar is 10 μm. h APF, hours after puparium formation.
(AVI)

S7 Movie Muscle laser cutting in a z-stack at 30 h APF. Movie of the *talin-I-YPet* pupa shown in Fig 4I. The black line indicates the position of the z-stack cut. Note that the muscle attachment recoils away from the cut. The length of the movie is 42.5 s with a time resolution of 5.3 s played at 20× speed. Scale bar is 10 μm. h APF, hours after puparium formation.
(AVI)

S1 Data All individual data points and statistical evaluation of the data shown in this manuscript. (XLSX)

## Abbreviations

C-F40-TS: Talin control sensor with F40-sensor module
C-mCh: Talin with C-terminal mCherry
C-stTS: Talin control sensor with HPst-sensor module
C-TS: Talin control sensor with HP-sensor module
C-YPet: Talin with C-terminal YPet
CPP: counts per particle
CRISPR/Cas9: clustered regularly interspaced short palindromic repeats/CRISPR-associated protein 9
FCS: fluorescence correlation spectroscopy
F40: Flagelliform peptide
F40-TS: Talin tension sensor with F40-sensor module
FCS: fluorescence correlation spectroscopy
FLIM: fluorescence lifetime imaging microscopy
FRAP: fluorescence recovery after photobleaching
FRET: Förster resonance energy transfer
h APF: hours after puparium formation
HP: Villin headpiece peptide
HPst: stable Villin headpiece peptide
I-mCh: Talin with internal mCherry
I-YPet: Talin with internal YPet
pN: piconewton
RNAi: RNA interference
RT: room temperature
sgRNA: single guide RNA
TCSPC: time-correlated single photon counting
stTS: Talin tension sensor with HPst-sensor module
*talin-IR*: *talin* RNA interference
TS: Talin tension sensor with HP-sensor module
WT: wild type
YPet: yellow fluorescent protein for energy transfer
