# Supplementary figures and images for: A small proportion of Talin molecules transmit forces at developing muscle attachments in vivo
Source: PLoS Biol. 2019 Mar 27;17(3):e3000057. doi: 10.1371/journal.pbio.3000057 (PMC6453563; doi:10.1371/journal.pbio.3000057)

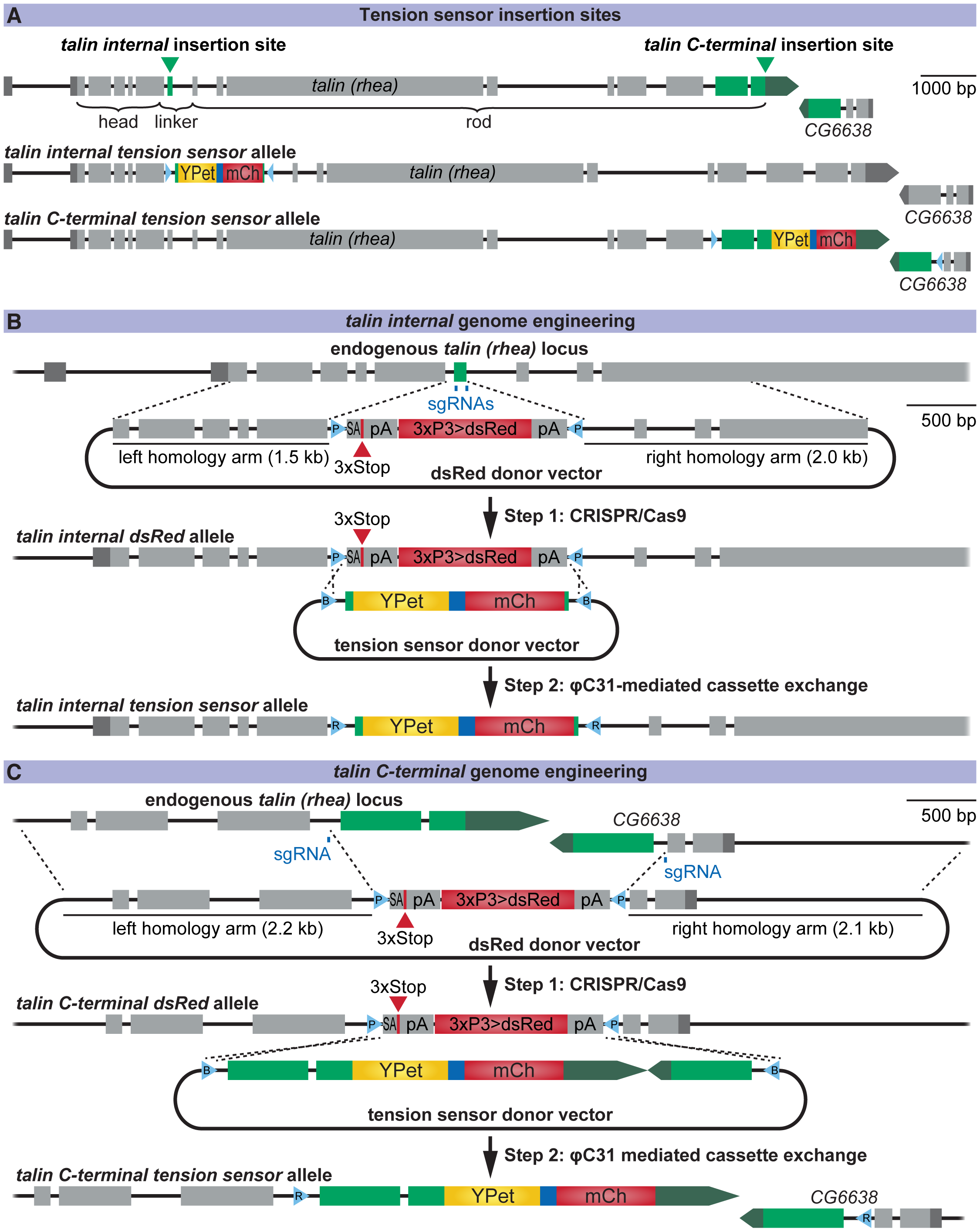

Supplement: S1 Fig — (A) Top: Gene model of talin (rhea, isoform RF) with the insertion sites (green) in the linker region between Talin head and rod (internal) and at the C-terminus. The gene CG6638 immediately follows talin and therefore was also included. Middle: Tension sensor allele with the sensor module inserted into the target exon in the linker region of Talin. attR sites left in the surrounding introns are shown in light blue. Bottom: C-terminal control sensor allele with the sensor module inserted at the C-terminus of Talin. Gene models are drawn to scale. (B) Scheme showing how tension sensor alleles were generated. Step 1: The target exon in the linker region (green) was replaced by a splice acceptor (SA)-3×Stop-SV40 terminator (pA)-3×P3>dsRed-pA cassette flanked by attP sites (P) using the CRISPR/Cas9 system. Specifically, a dsRed donor vector containing 1.5 to 2.0 kb homology arms was injected into Act5C-Cas9 expressing embryos (also carrying a DNAlig4169 mutation to favor homology-directed repair over nonhomologous end-joining [28]) together with 2 in vitro–transcribed sgRNAs (target sites in blue). Successful targeting was identified by screening for fluorescent red eyes. Step 2: ϕC31-mediated cassette exchange was performed to replace the dsRed cassette by the original target exon including a tension sensor module consisting of YPet, a flexible, calibrated, mechanosensitive linker peptide (dark blue), and mCherry (mCh). To this end, a tension sensor donor vector including flanking attB sites (B) was injected together with vasa-ϕC31 plasmid. Thereby, the tension sensor was inserted seamlessly into the gene (after Talin amino acid 456) except for 2 attR sites (R) in the flanking introns. Successful exchange events were identified by screening for the absence of fluorescent red eyes [28]. Control fly lines with 1 fluorophore and fly lines with different tension sensor modules were generated by repeating step 2 with different donor vectors. (C) Scheme showing how C-termina [file pbio.3000057.s001.tif]

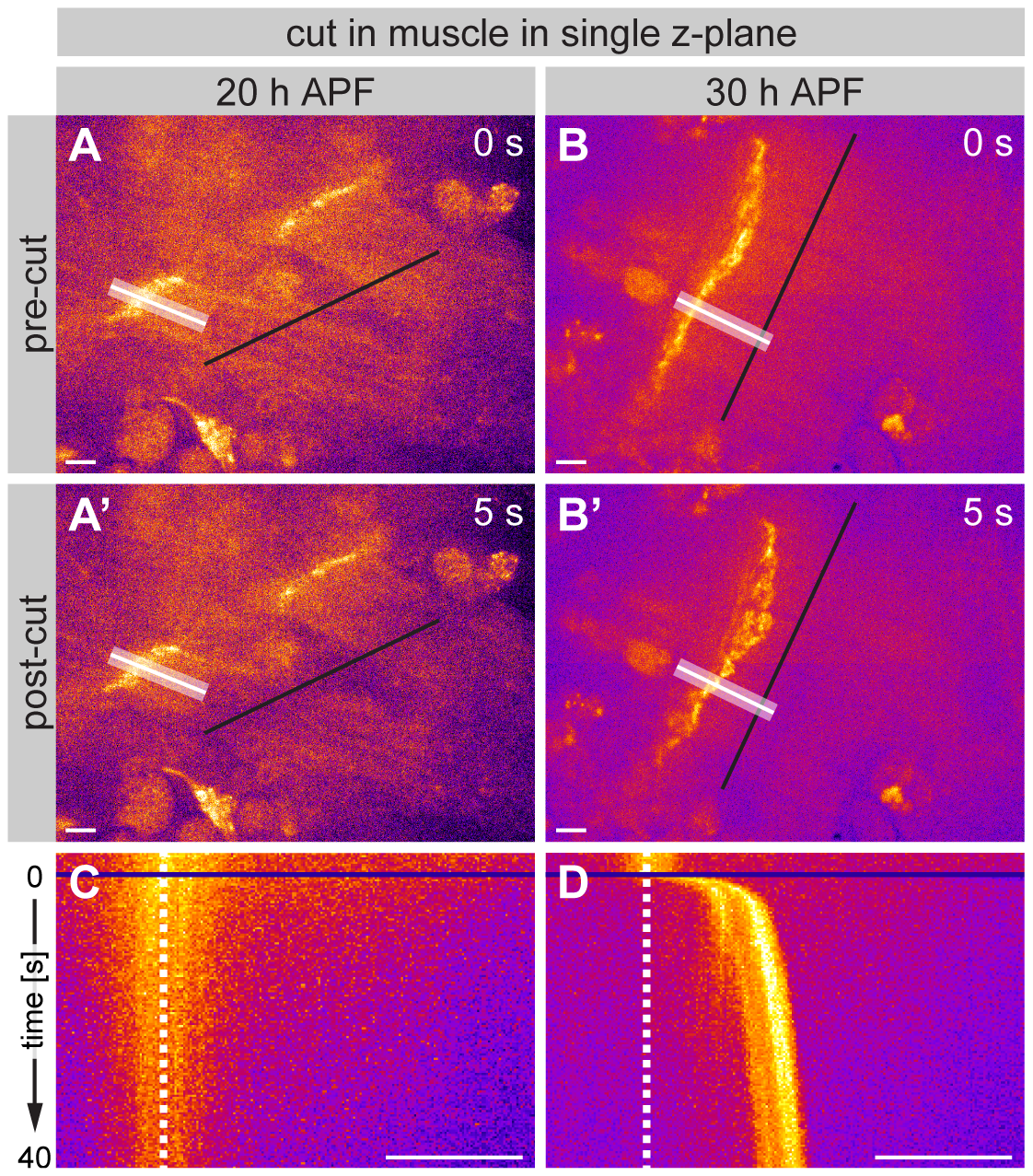

Supplement: S2 Fig — (A–B) Stills from movies of talin-I-YPet pupae before and after an incomplete cut of the muscle in a single z-plane. Black lines mark the position of the laser cuts, and white lines indicate areas analyzed in kymographs in C and D. (C–D) Kymographs showing muscle attachment movement. Dashed lines mark the position of the muscle attachments before the cut (at 0 s). Time resolution is 300 ms. Scale bars are 10 μm. See S4 and S5 Movies. h APF, hours after puparium formation. (TIF) [file pbio.3000057.s002.tif]

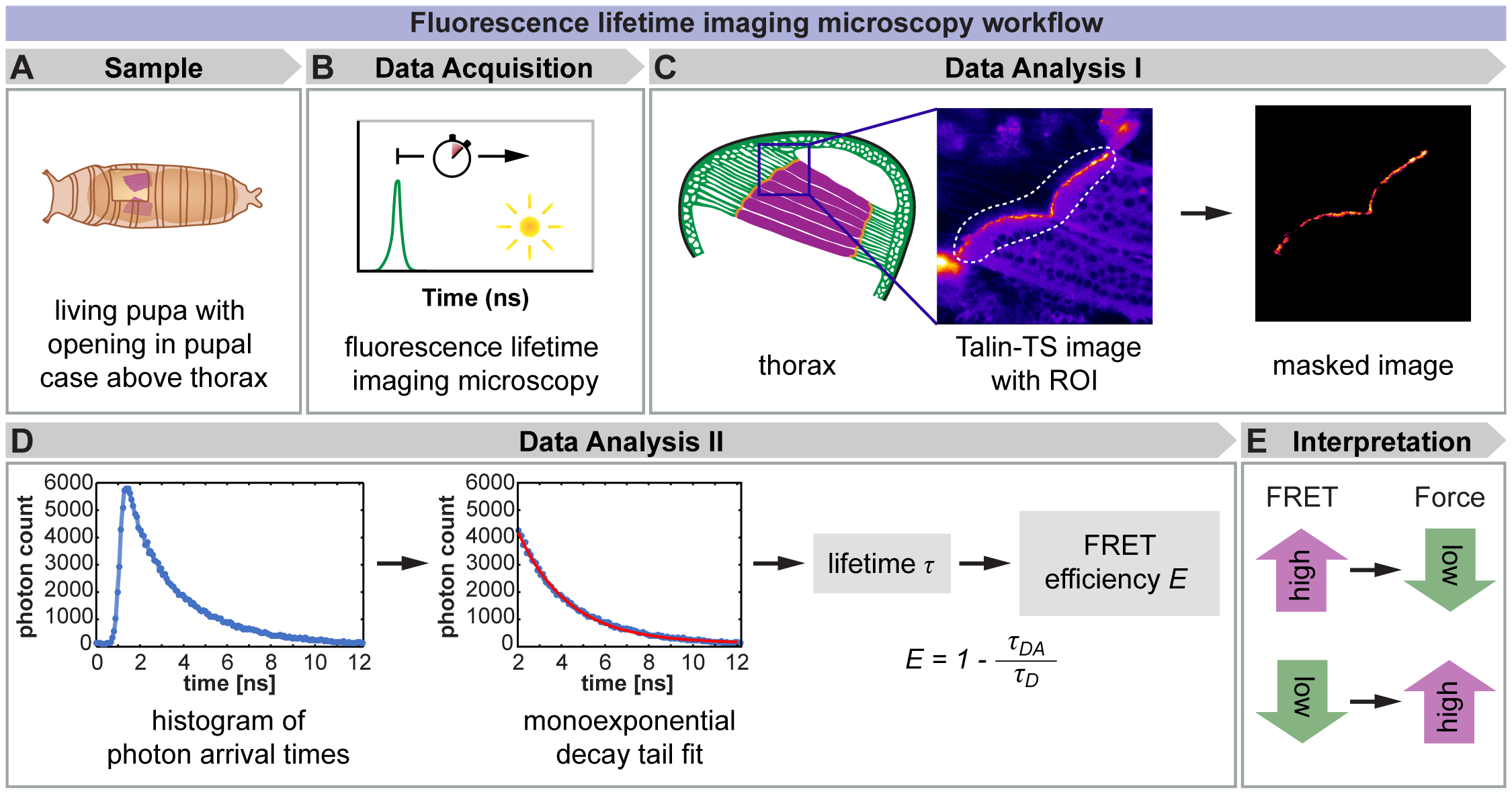

Supplement: S3 Fig — (A) Living talin-TS or control pupae were prepared for imaging by opening a window in the pupal case above the thorax containing the developing flight muscles (magenta) [61]. (B) FLIM was performed on a confocal microscope equipped with a pulsed laser (indicated by green peak) for exciting the donor fluorophore (YPet) and a TCSPC detector for recording photon arrival times (indicated by yellow dot). (C) A YPet intensity image created from the FLIM data was used to manually draw an ROI containing the anterior muscle attachments sites of the dorsal-longitudinal flight muscles close to the surface of the thorax. From this ROI, a mask for the muscle attachment sites was created by Multi-Otsu thresholding. (D) Photon arrival times of all photons inside the mask were plotted in a histogram. The tail of the curve was fitted by a monoexponential decay to determine the lifetime τ. By comparing the lifetime of the Talin tension sensor τDA with the lifetime of respective donor-only control τD, the FRET efficiency E was calculated. (E) Interpretation of FRET results: a high FRET efficiency indicates mostly closed sensor modules and therefore low force; vice versa, a low FRET efficiency indicates mostly open sensor modules and therefore high force. FLIM, fluorescence lifetime imaging microscopy; FRET, Förster resonance energy transfer; ROI, region of interest; TCSPC, time-correlated single photon counting; YPet, yellow fluorescent protein for energy transfer. (TIF) [file pbio.3000057.s003.tif]

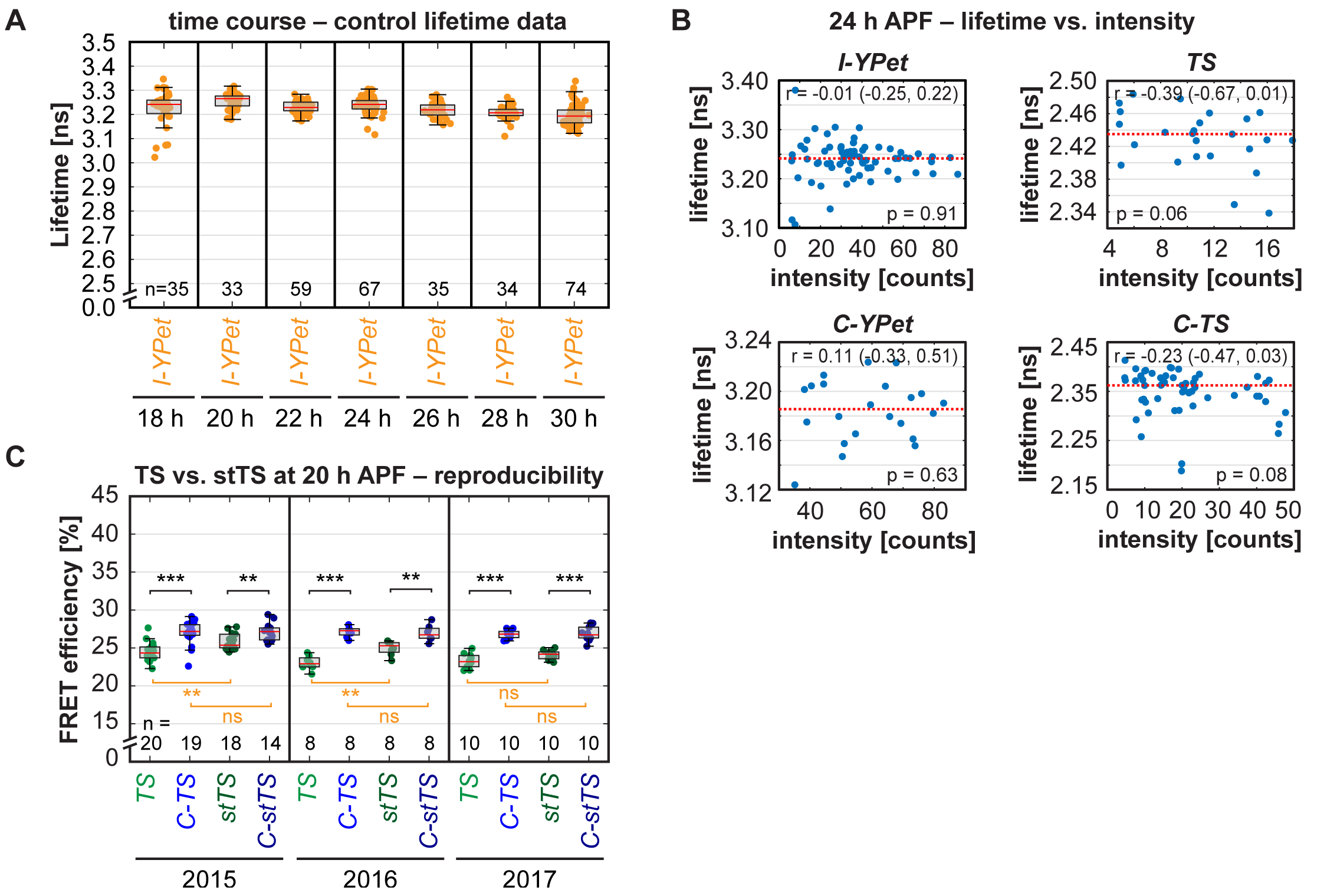

Supplement: S4 Fig — (A) Lifetime data of donor-only controls at the internal position of Talin (I-YPet) (B) Lifetime data for I-YPet, C-YPet, TS, and C-TS at 24 h APF for each pupa plotted against the average intensity inside its muscle attachment site mask. Red dotted line represents median lifetime value. No correlation between lifetime and intensity could be detected (Pearson correlation coefficient r with 95% confidence interval and p-values are indicated). (C) Reproducibility of FLIM-FRET measurements performed in different years: TS and its stable variant stTS show a reproducible decrease in FRET efficiency compared to the C-terminal zero-force controls C-TS and C-stTS at 20 h APF (Kolmogorov-Smirnov test, ***p < 0.001, **p < 0.01, ns: p > 0.05). Underlying data can be found in S1 Data. C-TS, Talin control sensor with HP-sensor module; C-stTS, Talin control sensor with HPst-sensor module; C-YPet, Talin with C-terminal YPet; FLIM, fluorescence lifetime imaging microscopy; FRET, Förster resonance energy transfer; h APF, hours after puparium formation; HP, Villin headpiece; HPst, stable Villin headpiece; I-YPet, Talin with internal YPet; ns, nonsignificant; stTS, Talin tension sensor with HPst-sensor module; TS, Talin tension sensor with HP-sensor module; YPet, yellow fluorescent protein for energy transfer. (TIF) [file pbio.3000057.s004.tif]

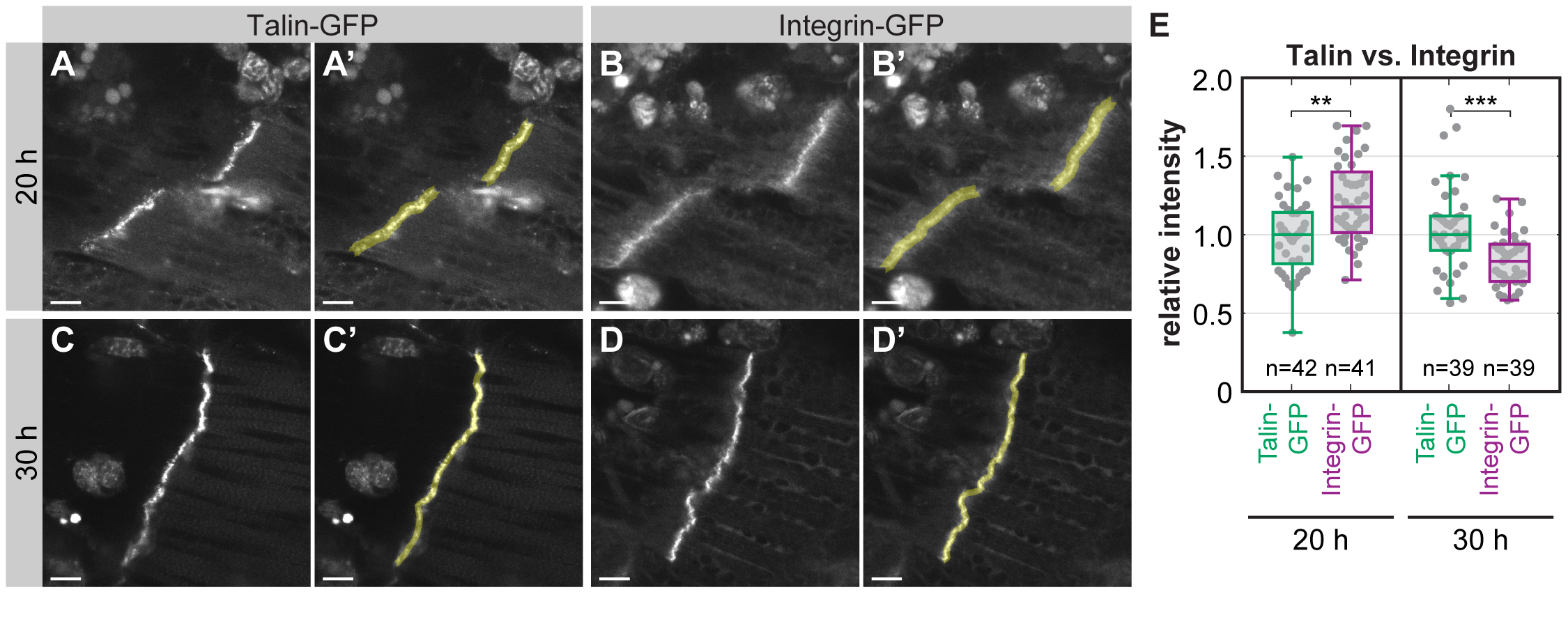

Supplement: S5 Fig — (A–D) Live imaging of flight muscle attachment sites at 20 h and 30 h APF. Pupae expressing Talin-GFP (A and C) were compared to pupae expressing Integrin-GFP (βPS-GFP) (B and D). Yellow shaded areas in A’–D’ indicate attachment regions in which GFP intensity was quantified. Scale bars are 10 μm. (E) Quantification of Talin- and Integrin-GFP intensity at muscle attachment sites. Median Talin-GFP intensity was set to 1 for 20 h APF and 30 h APF. (Kolmogorov-Smirnov test, ***p < 0.001, **p < 0.01). Underlying data can be found in S1 Data. GFP, green fluorescent protein; h APF, hours after puparium formation. (TIF) [file pbio.3000057.s005.tif]

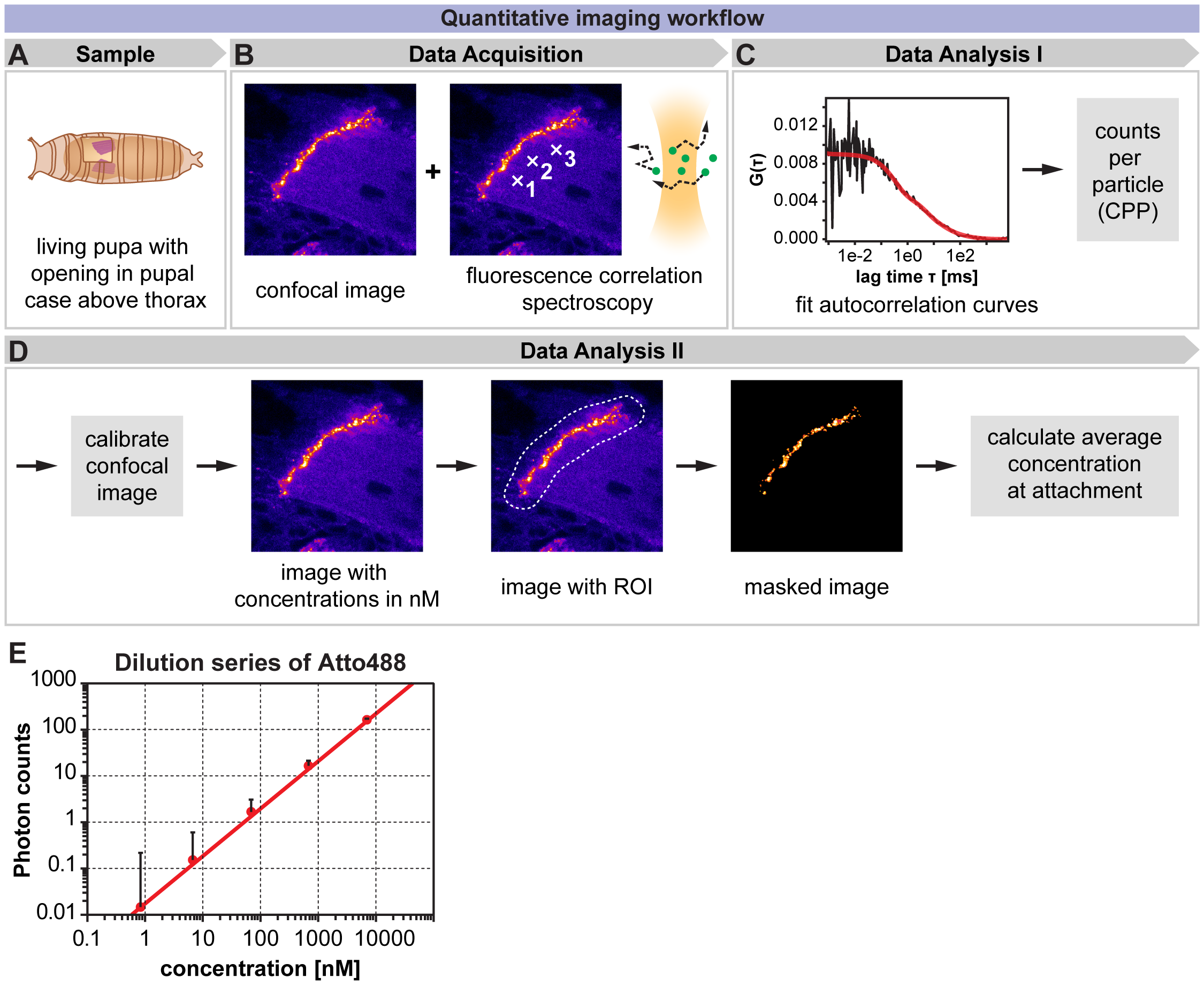

Supplement: S6 Fig — (A) Living talin-I-YPet pupae were prepared for quantitative imaging by opening a window in the pupal case above the thorax containing the developing flight muscles (magenta) [61]. (B) A confocal image and 3 FCS measurements were acquired using the same detector on a confocal microscope. (C) Autocorrelation curves from the FCS measurements were fit to obtain a CPP value for each pupa. (D) The CPP value was used to calibrate each image resulting in a pixel-by-pixel concentration image. This image was used to manually draw an ROI around the muscle attachment site. From this ROI, a muscle attachment mask was created automatically by Multi-Otsu thresholding. Finally, the average concentration at the attachment was calculated from the pixel values inside the mask for each pupa. (E) Pixel-by-pixel photon count values measured in confocal images of an Atto488 dye dilution series (mean with standard deviation). Note that the number of detected photons increases linearly with the concentration of the dye for the entire range measured. Thus, the high intensities at muscle attachment sites can be directly compared to low intensities in the muscle interior of the same confocal image. Underlying data can be found in S1 Data. CPP, counts per particle; FCS, fluorescence correlation spectroscopy; ROI, region of interest. (TIF) [file pbio.3000057.s006.tif]

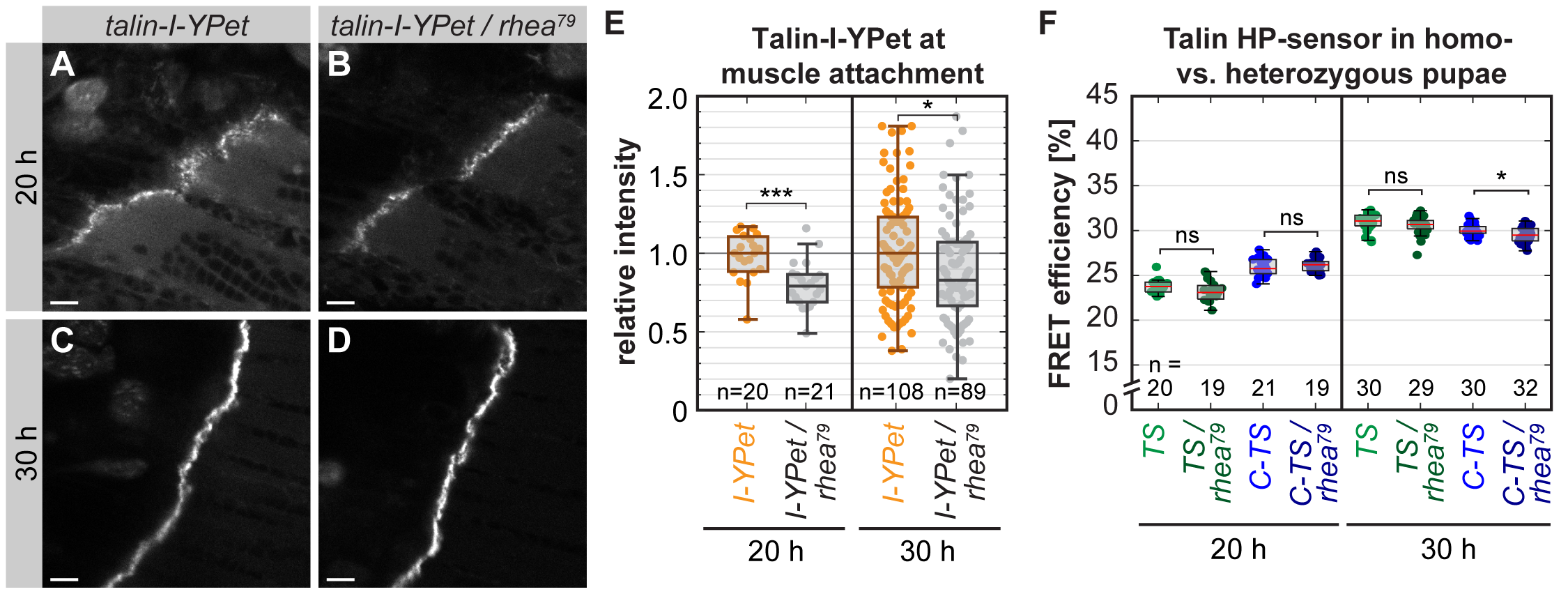

Supplement: S7 Fig — (A–D) Live imaging of flight muscle attachment sites of homozygous talin-I-YPet pupae (2 copies) and heterozygous talin-I-YPet/rhea79 pupae (1 copy). Scale bars are 10 μm. (E) Quantification of Talin-I-YPet (I-YPet) intensities at muscle attachment sites. Median intensity of homozygous pupae was set to 1 for 20 h and 30 h APF. Note that the Talin levels are only reduced to 80% and not 50% in heterozygous pupae. (F) Talin force measurements in homozygous and heterozygous tension sensor (TS) and zero-force control (C-TS) pupae. (Kolmogorov-Smirnov test, ***p < 0.001, *p < 0.05; ns: p > 0.05). Underlying data can be found in S1 Data. C-TS, Talin control sensor with HP-sensor module; h APF, hours after puparium formation; HP, Villin headpiece; I-YPet, Talin with internal YPet; ns, nonsignificant; rhea79, talin deficiency; TS, Talin tension sensor with HP-sensor module; YPet, yellow fluorescent protein for energy transfer. (TIF) [file pbio.3000057.s007.tif]

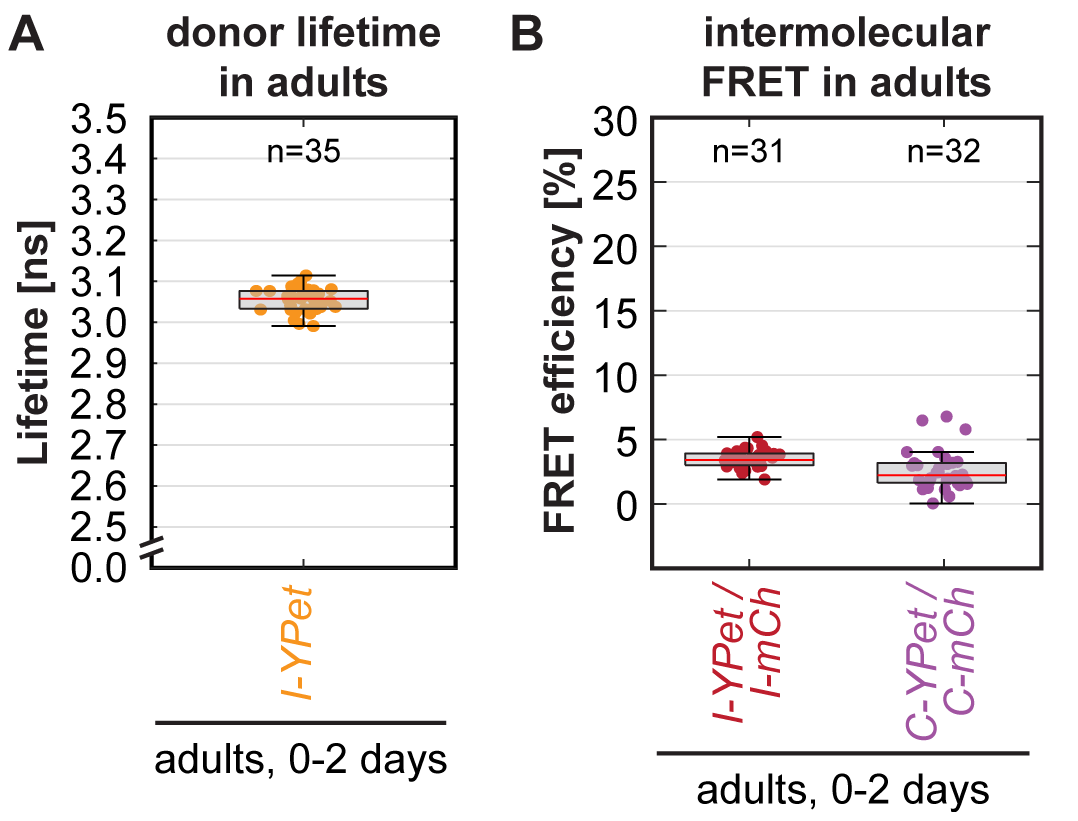

Supplement: S8 Fig — (A) Lifetime data of donor-only control at the internal position of Talin (I-YPet) in adults. The measured lifetime is slightly lower than in pupae at 18–30 h APF (S4A Fig), likely due to the short background lifetime of the adult cuticle that is just above to the muscle attachment sites. (B) Intermolecular FRET control data comparing heterozygous I-YPet/I-mCh or C-YPet/C-mCh pupae to homozygous I-YPet or C-YPet pupae, respectively. Underlying data can be found in S1 Data. C-mCh, Talin with C-terminal mCherry; C-YPet, Talin with C-terminal YPet; FRET, Förster resonance energy transfer; h APF, hours after puparium formation; I-mCh, Talin with internal mCherry; I-YPet, Talin with internal YPet; YPet, yellow fluorescent protein for energy transfer. (TIF) [file pbio.3000057.s008.tif]
